# Precursor types predict the stability of neuronal branches

**DOI:** 10.1242/jcs.258983

**Published:** 2021-12-06

**Authors:** Joachim Fuchs, Britta J. Eickholt

**Affiliations:** Charité – Universitätsmedizin Berlin, corporate member of Freie Universität Berlin, Humboldt-Universität zu Berlin, and Berlin Institute of Health, Institute of Molecular Biology and Biochemistry, Virchowweg 6, 10117 Berlin, Germany

**Keywords:** Neuron branch stability, PRG2, PLPPR3, Directed acyclic graphs, Survival analysis, Filopodium, Lamellipodium

## Abstract

Branches are critical for neuron function, generating the morphological complexity required for functional networks. They emerge from different, well-described, cytoskeletal precursor structures that elongate to branches. While branches are thought to be maintained by shared cytoskeletal regulators, our data from mouse hippocampal neurons indicate that the precursor structures trigger alternative branch maintenance mechanisms with differing stabilities. Whereas branches originating from lamellipodia or growth cone splitting events collapse soon after formation, branches emerging from filopodia persist. Furthermore, compared to other developing neurites, axons stabilise all branches and preferentially initiate branches from filopodia. These differences explain the altered stability of branches we observe in neurons lacking the plasma membrane protein phospholipid phosphatase-related protein 3 (PLPPR3, also known as PRG2) and in neurons treated with netrin-1. Rather than altering branch stability directly, PLPPR3 and netrin-1 boost a ‘filopodia branch programme’ on axons, thereby indirectly initiating more long-lived branches. In summary, we propose that studies on branching should distinguish overall stabilising effects from effects on precursor types, ideally using multifactorial statistical models, as exemplified in this study.

## Abstract (precis)

**Summary:** Branches of neurons differ in stability. We describe evidence that this stability is already defined during formation, suggesting separate branch maintenance modes initiated by distinct branch precursors.

## INTRODUCTION

Be they in rivers, lightning or trees – branches are ubiquitous in both receptive and transmitting processes in nature. Their abundance ensures optimal coverage of area, balancing maximal receptivity with the shortest distance to their origin. It is no surprise that branches are also used by neurons to optimise their signalling efficacy in a neuronal network. The cellular mechanisms controlling this specific branching behaviour have been studied for 30 years (summarised in Kalil and Dent, 2014). Recently, branching has gained further attention in the setting of regenerative growth of central nerve cells (Griffin and Bradke, 2020). Strategies to improve recovery following injury to the central nervous system include promoting branch formation in non-injured neurons to form alternative pathways (Fink et al., 2017) or inhibiting branching in injured neurons to facilitate undisturbed elongation (Tedeschi et al., 2019).

The main drivers of the remarkable morphology of neurons, the actin and microtubule components of the cytoskeleton, participate sequentially in branch formation. Initially, F-actin structures – filopodia or lamellipodia – remodel the plasma membrane. Subsequently, de-bundling, transport and polymerisation of microtubule arrays into the actin-enriched protrusion elongate the emerging branch. Not every branch will persist, however. Branches are maintained mainly by stabilising the microtubule cytoskeleton (Gallo, 2011; Kalil and Dent, 2014). Supporting this sequential model, F-actin regulators have been described to control branch emergence, whereas branch elongation requires crosslinkers between the two cytoskeletal structures, and branch maintenance is influenced predominantly by microtubule-associated proteins (Armijo-Weingart and Gallo, 2016).

Neurons establish networks by connecting selectively to specific regions and by establishing layer-specific receptive fields (Cuntz et al., 2010). Especially in long axons, this requires preventing most branching (Gibson and Ma, 2011). To circumvent the suppression of branching at specific sites, neurons rely on several extrinsic and intrinsic cues. One major regulator of branching, the phosphoinositide 3-kinase (PI3K)–PTEN pathway, triggers F-actin accumulation and protrusion formation (Kakumoto and Nakata, 2013; Ketschek and Gallo, 2010), induces transport and local translation (Akiyama and Kamiguchi, 2010; Spillane et al., 2013), and regulates microtubule stability (Kath et al., 2018). We previously described a transmembrane protein, phospholipid phosphatase-related protein 3 (PLPPR3; also known as plasticity-related gene 2, PRG2), that can relieve the general branch suppression by inhibiting PTEN, the negative regulator of PI3K signalling. Specifically, PLPPR3 redistributes growth towards branches by inducing filopodia formation (Brosig et al., 2019). Given that PI3K signalling is involved in multiple steps of branching, we set out to analyse the contribution of PLPPR3 to later stages, specifically to branch maintenance.

## RESULTS

### *Plppr3^−/−^* branches are less stable

**Fig. 1.**
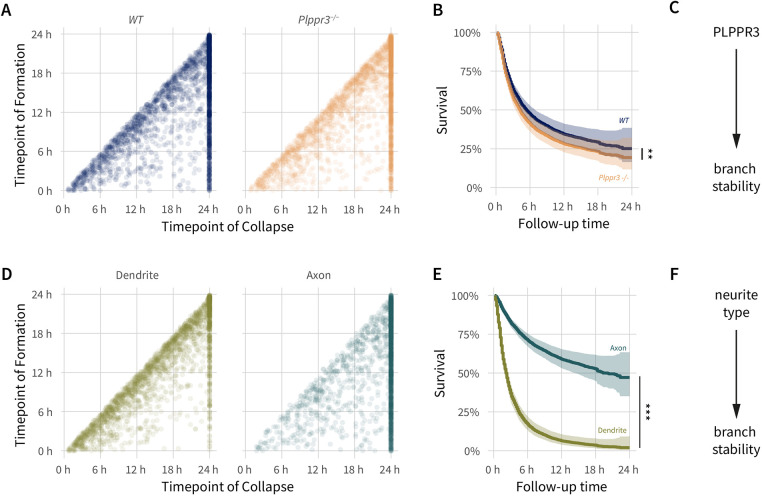
**Individual effects of PLPPR3 and neurite type on neuronal branch stability.** (A) Scatterplot of timepoint of formation versus collapse for individual branches shows strong right-censoring of the data for both WT and *Plppr3^−/−^* neuronal branches. (B) Correcting for censoring bias using survival analysis reveals a small effect of loss of *Plppr3* on branch lifetime. (C) Causal graph highlighting the connection of the *Plppr3* genotype to branch stability. (D) Scatterplot of timepoint of formation versus collapse for individual branches shows strong enrichment of non-collapsing branches in the axonal fraction of WT and *Plppr3*^−/−^ neurons. (E) Survival analysis shows a strong effect of the neurite type on branch stability in pooled data from both WT and *Plppr3^−/−^.* (F) Causal graph highlighting the connection of the neurite type to branch stability. *n*=2317 (WT) and 2165 (*Plppr3^−/−^*), *n*_experiments_=6 (six independent cultures). Shaded regions in survival curves show the 95% c.i. Survival analyses were analysed with Cox proportional hazards regression (**P<0.01; ***P<0.001).

We employed phase-contrast microscopy of cultured mouse neurons from wild-type (WT) and *Plppr3*^−/−^ hippocampus. Neurons were imaged for 24 h, at a temporal precision of 10-min intervals. In initial observations, *Plppr3^−/−^* branches appeared less stable (Movie 1, Fig. S1A). We therefore measured the lifetime of each branch as the difference between the time of initiation and, if applicable, collapse, in a blinded and randomised manner. However, such lifetime measurements depend strongly on the length of the observed time window and are extremely biased towards short lifetimes that collapse during observation. In our experiments, 40% of the branches did not collapse during the observed time window, even if they persisted for hours (heavily right-censored data, Fig. 1A).

Instead of raw lifetimes, we therefore quantified the risk for collapsing (the hazard ratio, HR) using Cox proportional hazard regression, a method commonly used in clinical trials to mitigate the effects of censoring in follow-up data (Leung et al., 1997). In such survival, or time-to-event analyses, HRs above 1 and decreased survival curves indicate an increased risk of branch collapse. This analysis established the *Plppr3* genotype as a predictor of branch stability (Fig. 1B,C; HR_*Plppr3*^−/−^_=1.2, 95% c.i.=1.1–1.3, *P*=0.006). Counterintuitively however, this effect is not evidence that PLPPR3 directly affects branch maintenance. Deeper analyses establish the effect of PLPPR3 on branch stability as secondary to its primary effect of inducing one specific branch precursor, filopodia (see below).

### Type of branch precursor predicts branch stability

During our analyses of branch stability in developing neurons, axonal branches appeared to persist longer than branches on immature dendrites (Movie 1, Fig. S1A). Supporting this, survival analyses of these branches distinguishing their neurite type (axon or immature dendrite) revealed a strong risk of collapse for branches on immature dendrites (Fig. 1D–F; HR_Neurite type_=5.2, 95% c.i.=4.6–5.9, *P*<0.001). This increased stability for axon branches is likely affected by mechanisms that govern stability of the newly polarised axon shaft. Here, microtubule stability has been observed to differ in developing axons and dendrites and even contributes to initial axon specification (Witte et al., 2008). Microtubule stability in turn is regulated by distinct microtubule-binding proteins in axons (reviewed in Conde and Cáceres, 2009) and correlates well with differences in orientation and post-translational modifications of microtubules (reviewed in Janke and Magiera, 2020).

**Fig. 2.**
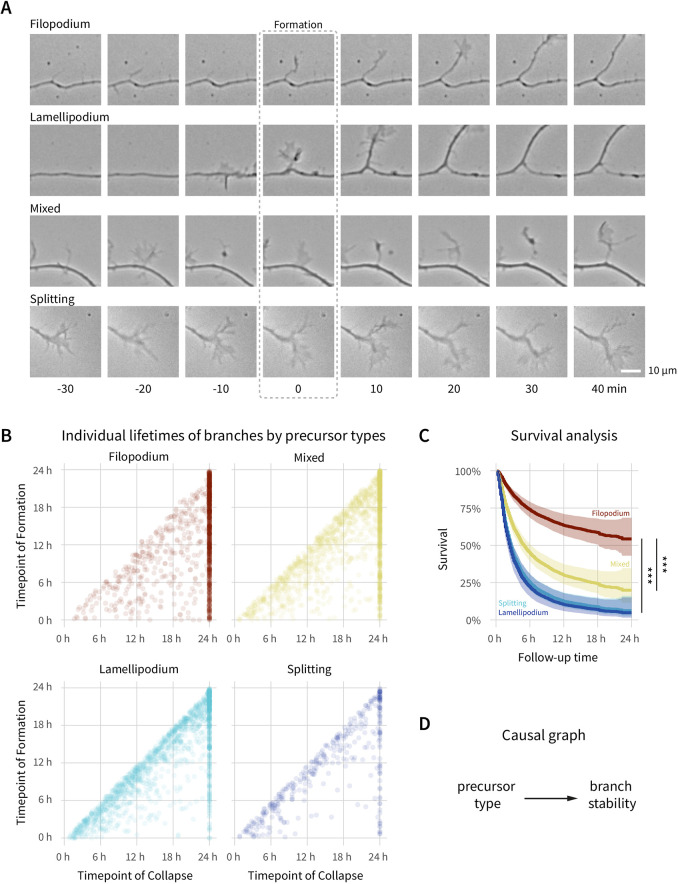
**Morphologically distinct branch precursor types affect branch stability.** (A) Example time series of branch precursor types. Indicated times are relative to the timepoint of branch formation (0 min). (B) Scatterplot of timepoint of formation versus collapse for individual branches shows strong right-censoring of filopodia-associated branches when compared to those associated with other branch precursors. (C) Survival analysis shows the strong effect of the branch precursor type on stability in pooled data of both WT and *Plppr3^−/−^*, with filopodia initiating the most stable branches. (D) Causal graph highlighting the connection of precursor type to branch stability. *n*=2317 (WT) and 2165 (*Plppr3^−/^*^−^), *n*_experiments_=6 (six independent cultures). Shaded regions in survival curves show the 95% c.i. Survival analyses were analysed with Cox proportional hazards regression (***P<0.001).

To our surprise, however, the type of F-actin-based branch precursor also strongly influenced subsequent branch stability. We classified branches by the morphology of their precursor types (Fig. 2A) as bifurcations of the growth cone (‘splitting’) or as formations on the axon shaft (collateral branches) originating from thin filopodia or sheet-like lamellipodia. Because many collateral branches initiating from filopodia are invaded by lamellipodia directly before branch elongation (Flynn et al., 2009; Withers and Wallace, 2020), we added a hybrid class (‘mixed’). Our quantification revealed that branches originating from lamellipodia and growth cone splitting were at a high risk of collapse within a few hours (HR_Lamellipodia_=4.6, 95% c.i.=3.6–5.7; HR_Splitting_=5.0, 95% c.i.=3.8–6.6; both *P*<0.001), whereas most branches originating from filopodia remained stable throughout the 24 h imaging period (Fig. 2B–D). Mixed precursor branches were at an intermediate risk of collapse (HR_Mixed_=2.6, 95% c.i.=2.1–3.3, *P*<0.001). While lamellipodia-associated branches appear to be stochastic ‘trial-and-error’ branch initiations, filopodial branches may be a deterministic mode that form to stay.

### Axons initiate similar numbers of branches but use different precursors than immature dendrites

**Fig. 3.**
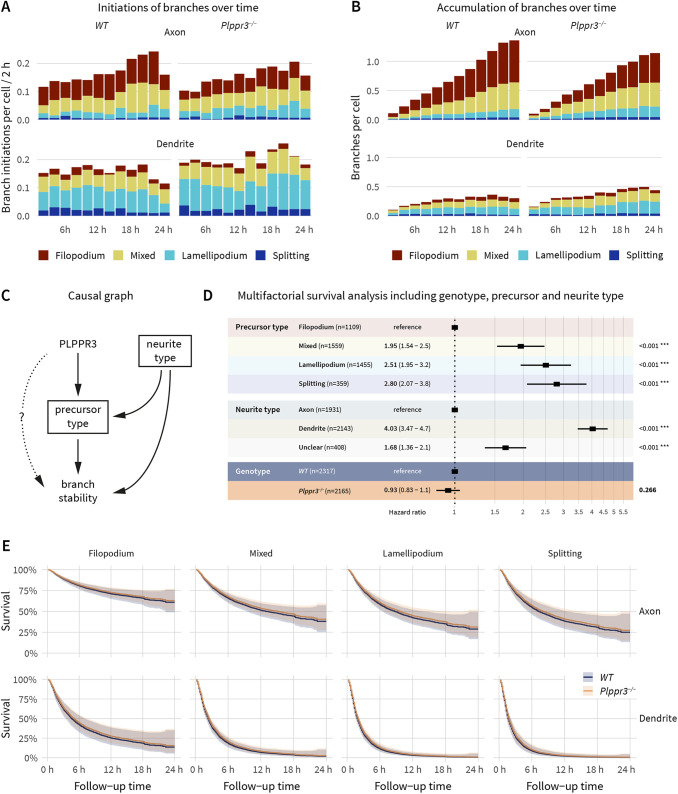
**Combined influences on branch stability.** (A) Distribution of branch-forming events over time, colour-coded by precursor type, according to neurite type as well as genotype. Timepoints are binned in 2-h windows. Note the similar overall number of branch initiations but different composition of precursor types between axons and immature dendrites. (B) Branches per cell present over time, colour-coded by precursor type, according to neurite type as well as genotype. Note the increased branch accumulation on axons versus immature dendrites and the different precursor composition between branch initiations (A) and accumulating branches (B). Bin size 2 h. (C) Directed acyclic graph aggregating the information from Figs 1C,F, 2D and 3A, as well as Fig. S1B. According to causal graph theory, to quantify a direct effect of PLPPR3 on branch stability (dotted line), a multifactorial analysis has to adjust for precursor type as well as neurite type (indicated by squares). (D,E) Forest plot (D) and survival curves (E) of a Cox proportional hazard analysis of WT versus *Plppr3^−/−^* branches including neurite type as well as precursor types as covariates demonstrates no direct effect of PLPPR3 on branch stability. HR values and the 95% c.i. are listed in D. Boxes in D indicate the HR, lines indicate the 95% c.i., and *P*-values are shown on the right (***P<0.001). *n*=2317 (WT) and 2165 (*Plppr3^−/−^*), *n*_experiments_=6. The shaded regions in E show the 95% c.i. of the survival curves.

Similar to previous studies (Armijo-Weingart and Gallo, 2016), we found that neurons in our experimental setup initiated most branches as collaterals, with comparable numbers of lamellipodial, filopodial and ‘mixed’ precursor branches, whereas bifurcations were scarce (Fig. 3A; Fig. S1B). Furthermore, while the total number of branch initiation events was similar between axons and immature dendrites (Fig. 3A), the proportions of the precursor types differed drastically, with filopodia mainly dominating axon branch initiations and lamellipodia being the predominant branch precursor of immature dendrites. This difference became even more apparent over time, when visualising which precursor type the branches originated from (Fig. 3B). Most branches on axons originated from filopodia and mixed precursors and accumulated quickly, whereas branches on immature dendrites hardly accumulated, irrespective of the precursor type. In addition to using the most efficient precursors, axons therefore also seem to stabilise branches of all precursors.

Compared to WT neurons, *Plppr3^−/−^* neurons initiated fewer branches from filopodia precursors (Fig. 3A; Fig. S1B; *P*=0.046), without affecting initiations from other precursor types. *Plppr3^−/−^* neurons also seemed to accumulate fewer branches on axons (Fig. 3B). This is in accordance with our recent study that reported a lower number of filopodia in *Plppr3*^−/−^ cells, specifically on early polarised axons, and a lower number of axon branches at later developmental stages (Brosig et al., 2019). Notably, the effect sizes of filopodia density, branch density (figure 6 in Brosig et al., 2019) and branch initiations from filopodia (this study) are very similar. This indicates that the initial filopodia number is the main determinant of the branching defect in *Plppr3^−/−^* neurons rather than a defect in filopodia-to-branch transitions.

### PLPPR3 regulates branch stability via the precursor type alone

We showed that loss of PLPPR3 decreases branch stability (Fig. 1B,C) and reduces the numbers of the most efficient precursor, filopodia (Fig. S1B; Brosig et al., 2019). PLPPR3 does so preferentially on the axonal compartment of neurons (Brosig et al., 2019), which itself stabilises branches (Fig. 1E,F) and utilises more efficient precursors (Fig. 3A). With such individual but interdependent information it is difficult to distinguish whether the PLPPR3 effect on branch stability is direct, as is conceivable by inducing PI3K signalling and microtubule stability, or indirect, by regulating the number of filopodia.

This information, however, helps to generate an informed causal graph [directed acyclic graph (DAG); Pearl, 1995], which in turn can form the basis of a multifactorial statistical analysis quantifying the contribution of the various effects. A causal graph summarising these data (Fig. 3C) assumes that both precursor and neurite type directly influence branch stability, and that PLPPR3 directly influences the abundance of one precursor type. Furthermore, the neurite types differ in the distribution of precursor types. According to causal graph theory (Pearl et al., 2016), to determine in this setting whether there is a direct effect of PLPPR3 on branch stability (dotted line in Fig. 3C), an analysis has to adjust for the precursor type as well as neurite type to obtain an unbiased estimate.

After adjusting our survival analysis for the contribution of precursor and neurite type, the stability of *Plppr3^−/−^* branches was indistinguishable from that of WT branches both when inspecting the HR (Fig. 3D; HR=0.9 95% c.i.=0.8–1.1; *P*=0.26) and the individual survival curves of all precursor type and neurite type combinations (Fig. 3E). This still means that *Plppr3^−/−^* branches are less stable than WT branches (Fig. 1B,C); however, there is no evidence in this dataset for a direct effect of PLPPR3 on branch stability. The effect of PLPPR3 on branch stability is fully explainable as an indirect consequence of its effect on filopodia abundance.

### Other branch-inducing factors differ in their effect on branch stabilisation

The analyses of the presented dataset reveal that the stability of a branch is predicted by its emergence from specific precursor types. Furthermore, they suggest that altering the precursor type composition by reducing the number of filopodia-induced branches, with loss of PLPPR3, can indirectly affect branch stability. To test whether this effect of branch precursors on branch stability generalises to other modifications of precursor types, we induced branch formation through two independent treatments. Both guidance cues (such as netrin-1) and growth factors (such as FGF-2) increase overall branch numbers (reviewed in Kalil and Dent, 2014). While netrin-1 has been described to induce filopodia (Dent, 2004; Szebenyi et al., 2001; Winkle et al., 2014), FGF-2 appears to additionally involve growth cone and lamellipodial effects (Dent, 2004; Dos Santos et al., 2019; Szebenyi et al., 2001). To assess whether these treatments can alter branch stability by their effects on precursor types, we treated WT neurons with netrin-1 or FGF-2 for 1 hour before recording branching behaviour for 30 h and analysing as described above.

**Fig. 4.**
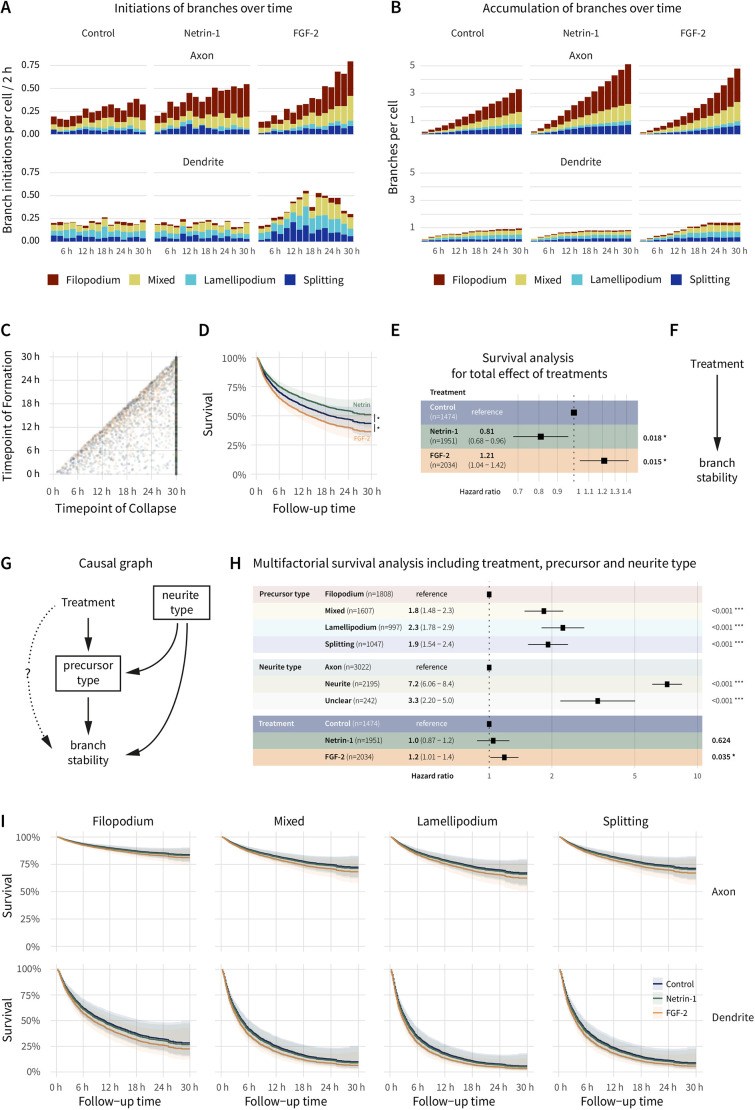
**Netrin-1 increases branch stability via the precursor type and FGF-2 decreases branch stability in addition to the precursor type.** (A) Distribution of branch-forming events over time, colour-coded by precursor type, according to neurite type as well as treatment. Note the effects of FGF-2 on both axons and dendrites and the overall increase in branch inductions over time. Bin size 2 h. (B) Branches per cell present over time, colour-coded by precursor type, according to neurite type as well as treatment. Note the increased branch accumulation after netrin-1 and FGF-2 treatments. (C) Scatterplot of timepoint of formation versus collapse for individual branches from all treatment groups (control, blue; netrin-1, green; FGF-2, orange) shows strong right-censoring of branch lifetime data in an independent experiment, even after 30 h. (D,E) Correcting for censoring bias using survival analysis reveals small but opposite effects of netrin-1 (green) and FGF-2 (orange) on branch stability compared to that of the control (blue). HR values and the 95% c.i. are listed in E. Boxes in E indicate the HR, lines indicate 95% c.i. and *P*-values are shown on the right. Survival analyses were analysed with Cox proportional hazards regression. (F) Causal graph highlighting the total effects of treatments on branch stability. (G) Directed acyclic graph for testing precursor-type-independent effects (dotted line) of the treatments on branch stability. As in the previous experiment, a multifactorial analysis has to account for precursor type as well as neurite type (indicated by squares). (H) Forest plot of a Cox proportional hazard analysis of control versus netrin-1- or FGF-2-treated branches including neurite type as well as precursor types as covariates demonstrates no direct effect of netrin-1 on branch stability but more complex effects of FGF-2. Note how the HR of netrin-1 treatment changes compared to the analysis in E, whereas the HR of FGF-2 is not affected. HR values and the 95% c.i. are listed. Boxes indicate the HR, lines indicate 95% c.i. and *P*-values are shown on the right. (I) Survival curves of the analysis in H. Note the near-perfect match of control and netrin-1 survival curves and the slight difference of the FGF-2 curve. All data presented in this figure describe three independent experiments of WT hippocampal neurons treated with DMSO, netrin-1 or FGF-2. *n*=1474 (control), 1951 (netrin-1), 2034 (FGF-2) *n*_experiments_=3. Shaded regions in D and I show the 95% c.i. of the survival curves. *P<0.05; ***P<0.001.

The results show that both netrin-1 and FGF-2 increased initiations (Fig. 4A; Movie 2, Fig. S2A) as well as accumulation of branches on axons, verifying successful treatment conditions. FGF-2 treatment additionally resulted in strong branch inductions on immature dendrites (Fig. 4B). Whereas netrin-1 seemed to predominantly affect branch inductions from axonal filopodia, FGF-2 appeared to increase inductions from all precursor types without changing the overall composition of precursors (Fig. 4A,B; Fig. S2B). Interestingly, both netrin-1 and FGF-2 affected branch stability (Fig. 4C–F). While netrin-1-induced branches were more stable (HR_netrin-1_=0.81, 95% c.i.=0.68–0.96, *P*=0.018), FGF-2-treated branches were at a higher risk of collapse (HR_FGF-2_=1.21, 95% c.i.=1.04–1.42, *P*=0.015).

Given the observed and published effects of netrin-1 and FGF-2 on precursor types (Dent, 2004), the causal graph for this comparison is very similar to the graph describing loss of PLPPR3 (Fig. 4G). As before, the graph can be used to specify a multifactorial survival analysis to estimate the effects on branch stability that are not mediated by altered precursor type compositions. Interestingly, in such a controlled survival analysis (Fig. 4H,I), the netrin-1 effect on branch stability was fully explained by its effect on precursor types (HR_netrin-1_=1.0, 95% c.i.=0.87–1.2, *P*=0.642), as was the case for PLPPR3. In contrast, the HR for FGF-2-treated branches was not altered by accounting for precursor and neurite type (HR_FGF-2_=1.2, 95% c.i.=1.01–1.4, *P*=0.035).

This independent dataset corroborates the importance of studying precursor types when assessing branch stability. The analysis suggests a fully explainable effect of netrin-1 on branch stability by modifying precursor type compositions. In contrast to this, FGF-2 appears to induce a more complex branching phenotype, both initiating more branches while at the same time decreasing their stability.

## DISCUSSION

### Branch precursor types initiate distinct branching systems

Our analyses in hippocampal neurons highlight a strong influence of the neurite type as well as the precursor type on branch stability. We identified that filopodia are not the most abundant precursor but are the most efficient. Losing filopodia-initiated branches (in *Plppr3*^−/−^ neurons) appears to decrease the stability of the remaining branches, whereas a recent study has suggested that specifically losing lamellipodia results in a trend towards increased branch stability in a sample of only three neurons (Pollitt et al., 2020). Furthermore, increasing filopodia-initiated branches by netrin-1 application increases the stability of branches. In contrast to FGF-2, netrin-1 or loss of PLPPR3 appear to shift the equilibrium of branch precursor types without affecting the stability of branches from each individual precursor. This suggests that the different precursors initiate distinct types of branches with mechanistically independent maintenance programmes.

This raises the question of how the configuration of the actin cytoskeleton in the branch precursor type influences branch stability hours after the precursor structures elongate. The mechanical rigidity of parallel-bundled F-actin in filopodia and the meshwork of F-actin in lamellipodia may account for differences in branch stability, as forces generated by filopodia and lamellipodia in growth cones differ (Cojoc et al., 2007), and microtubule growth is receptive to force (Janson et al., 2003). In this respect, it could be interesting to test whether long-lived precursors are more likely to induce long-lived branches or not.

Alternatively, branch precursors may recruit different actin–microtubule crosslinkers (Dogterom and Koenderink, 2019) for microtubule capture in forming branches. Interesting candidates for filopodia-induced branches include septin 7 and drebrin, which both participate in initial phases of microtubule invasion and localise in filopodia (Hu et al., 2012; Ketschek et al., 2016). Alternatively, precursor types could use different mechanisms to supply fresh microtubules via severing, transport or *de novo* nucleation. Differential modes of branch initiation have been described for the microtubule-severing enzymes katanin and spastin (Yu et al., 2008). Most importantly, branches from different precursors can be expected to differ in the microtubule-stabilising factors they recruit. MAP7 (Tymanskyj and Ma, 2019; Tymanskyj et al., 2017), MAP7D2 (Pan et al., 2019), MAP6 (Tortosa et al., 2017) and the endoplasmic reticulum (Farías et al., 2019) have recently emerged as fruitful candidates to either mediate precursor-specific or global axon-associated effects on branch stability.

Microtubules and their binding proteins, however, can also actively modify the F-actin-based branch precursors. Lamellipodial actin waves both co-occur with and require dynamic microtubules (Winans et al., 2016). The microtubule-binding proteins doublecortin (DCX; Fu et al., 2013) and GAS2L1 (van de Willige et al., 2019) regulate F-actin stability, altering axon branching. Reduction of MAP7 levels induces more branch initiations while decreasing branch stability (Tymanskyj and Ma, 2019), which might be explained by the induction of more lamellipodia branches or by separate effects on microtubule stability and precursor types.

Our results presented here furthermore inform on the commonalities and differences of axonal and dendritic branching. Both developing axons and dendrites have been described to initiate branches from all precursor types (Armijo-Weingart and Gallo, 2016; Gascon et al., 2006; Heiman and Shaham, 2010; Korobova and Svitkina, 2010). However, developing axons and dendrites differ dramatically in their growth and (unsurprisingly also) their branching patterns, and treating their branching programmes as identical masks important differences. For this reason, many studies assess branching in an axon- or dendrite-specific manner. While this strategy allows for detection of differences between branching mechanisms, analysing them as completely unrelated processes complicates inferences about shared parts of the physiology.

The presented multifactorial analyses suggest that both developing axons and dendrites utilise all precursor types and that the precursor types predict branch stability on immature dendrites as well as on axons. The analyses further suggest that axons predominantly initiate branches from efficient precursors like filopodia, while lamellipodia-associated initiations are more common on dendrites. This distinct composition of precursor types does, however, not fully explain the differences between axon and dendrite branch stability in both presented multifactorial analyses, indicating that distinct mechanisms in axons stabilise branches irrespective of the precursor types. Given that the developing axon seems to preferentially use efficient precursors and to stabilise all branches irrespective of precursors in these datasets of developing neurons, future studies on branch-stabilising factors should distinguish effects on all branches from those on specific precursors.

### Cell biology can benefit from multifactorial analyses informed by causal models

In addition to the biological findings, this study also highlights how interpreting the effects of multiple interdependent factors independently can misinform the mechanistic models inferred from data, often establishing more pathways than the data accounts for. While cell biology routinely uses statistical tests to protect against false positive findings in individual experiments, the integration of evidence from multiple sources by collecting and discussing data (for example in scientific reviews) does not formally test their relationships. Consequentially, resulting models often contain more connections than are experimentally verified.

Our work highlights the value of quantifying the relationship of individually published links in additional, multifactorial experiments. Fortunately, statistical (multiple regression) and causal tools (DAGs, counterfactuals) have evolved considerably (Hernán and Robins, 2020; Rohrer, 2018; Suttorp et al., 2015). They are frequently employed in other fields, such as epidemiology (Greenland et al., 1999) or ecology (Greenacre and Primicerio, 2013; James and McCulloch, 1990), to reduce the number of false positive links in multifactorial systems.

Our study exemplifies how multifactorial statistical analyses informed by causal graphs can advance cell biology, offering a clear benefit over unifactorial ANOVAs and *t*-tests, and how leveraging this methodological approach has the potential to clarify the structure of biological pathways.

## MATERIALS AND METHODS

The experimental strategies are summarised in Fig. S3, and a detailed description of each step is provided below. Resources used in this study are summarised in Table S1.

### Animal procedures and primary neuron culture

Mice were housed and handled according to local ethical guidelines and approved animal care protocols (under the licence T0347/11, Landesamt für Gesundheit und Soziales Berlin) according to the guidelines of the animal welfare of Charité Universitätsmedizin Berlin. The mice were housed in standardised conditions under a 12-h day–night cycle, with water and food available *ad libitum*.

The *Plppr3^−/−^* line (described in Brosig et al., 2019) is maintained in a C57Bl/6 NCrl background. Heterozygous parents were bred for primary neuron culture preparation from day 16.5 embryos. Briefly, hippocampi of homozygous littermates (WT or knockout) were pooled for further single cell isolation without stratifying by sex. Extracellular matrix was degraded for 15 min using 10% trypsin in HBSS (Life Technologies), and samples were washed with HBSS with 1% horse serum (Sigma) and Neurobasal A medium (Life Technologies) before trituration to single cells using glass pipets.

Four-well glass-bottom chamber slides (µ-Slide, Ibidi) were coated sequentially with laminin (20 mg/ml, Sigma) and poly-ornithine (15 mg/ml, Sigma) before plating hippocampal neurons at a density of 25,000/cm^2^ in Neurobasal A medium containing 2% B27 (Life Technologies), 1% penicillin–streptomycin (Life Technologies), 100 mM β-mercaptoethanol (Applichem) and 1% GlutaMAX (Life Technologies). Neurons were grown at 37°C and 5% CO_2_ in a humidified incubator for 48 h before starting live-cell imaging. Netrin-1 (100 ng/ml, R&D Systems) and FGF-2 (20 ng/ml, PeproTech) treatments were applied in the microscope setup 1 h before imaging to not mechanically disturb imaging. Each individual neuron culture was considered as an independent *N*. The sample size was not estimated via power analysis prior to experiments due to lack of effect size estimates for the question under study. Instead, we chose the sample size to exceed typical sample sizes in cell biological experiments.

### Long-term live-cell microscopy

Long-term live-cell recordings were undertaken with a Nikon Eclipse Ti microscope equipped with a small stagetop as well as full incubator enclosure, to maintain cells at 37°C (in the full incubator) and 5% CO_2_ and humidity (in the stagetop) throughout the imaging session. Growing hippocampal neurons were visualised using Köhler adjusted phase contrast (Ph2) brightfield microscopy in areas of similar density across conditions using Nikon's Perfect Focus System to adjust for thermal fluctuations in focus. Three fields of view per genotype per litter were imaged for 24 h in 10-min intervals resulting in 18 movies from six cultures per group.

### Manual classification of branch events

Prior to analysis, movies were randomised and renamed automatically using a custom R script (Randomize_folder.R; https://github.com/jo-fuchs/Microscopy_analysis_snippets/tree/master/R) to perform the analysis blind to genotypes. For each movie, the total number of cells was recorded to adjust for differences in density between cultures. At each timepoint, newly forming branches (defined as processes longer than 10 µm) were marked with a region-of-interest (ROI) overlay using the FIJI ImageJ ROI manager (Schindelin et al., 2012). The line colour of the ROI was encoded to represent four morphologically distinct precursor types – growth cone splitting (blue), filopodium (red), lamellipodium/actin wave (green) and a mixed type (yellow).

In a second round of analysis, branch type classifications were quality controlled and for each branching event the neurite type was recorded as: (1) on an immature dendrite of both polarised and non-polarised neurons or (2) on the axon of clearly polarised neurons, defined as the persistently longest process of a neuron. Branching events on processes with cell bodies outside of the field of view were classified as ‘unclear’. Additionally, the timepoint of collapse for each branch was recorded as the time at which a branch – or the originating process – completely retracted. The timepoint of collapse for branches that did not collapse during the recordings was set to the last frame and treated as censored data in subsequent analyses. All ROIs were saved and exported as comma separated value (csv) files named correspondingly to the movie.

### Statistical analysis of branch initiation and lifetime

All further analysis steps were performed in R/RStudio (R Core Team, 2020) and are fully documented at https://github.com/jo-fuchs/Branch-Lifetime-PRG2. Briefly, individual branch event and cell count files were merged and unblinded (merge_data.R) before merging the cell count with the individual branching data. Further cleaning steps (clean_data.R) included converting frame counts to hours, calculating lifetime, creating a censoring indicator (whether a branch was present in the last frame) and calculating inverse probability weights (1/time until end of movie) to correct for the higher risk of censoring for branches forming close to the end of the data acquisition.

All statistical analyses are described in the R-scripts used to create the figures (Figure_1.R, Figure_2.R, Figure_3.R). Branch formations per cell (Figs S1B and S2B) were summarised per experiment. Assumptions for linear models were tested graphically using residual versus fitted, Q–Q, scale-location and Cook's distance plots and by Levene and Shapiro–Wilk tests on residuals. Welch's *t*-test was used for post-hoc pairwise comparisons. In cases of more than two comparisons, *P*-values were adjusted using Holm's correction.

For the branch incidence (Figs 3A and 4A) and branch accumulation (Figs 3B and 4B) over time, branching and collapse events were binned into 2-h slots (cumulative_branches.R). For each bin, the net ‘flux’ of branches (formed branches minus collapsed branches) was determined stratified by neurite type, precursor type and genotype. The accumulation of branches was determined by the cumulative sum of this ‘flux’, normalised by the number of cells the branches originated from.

Survival analyses of branch lifetimes were computed using Cox proportional hazards models including individual factors (Figs 1B,E, 2C and 4D,E) or the full models presented in Figs 3D,E and 4H,I. Assumptions were tested using Schoenfeld's test and inspecting residual plots, and all models were weighted by the inverse probability weight calculated above to account for the higher chance of censoring for branches forming close to the end of the imaging session. Final styling of the figures was performed in Adobe Illustrator 2021.

## Supplementary Material

Supplementary information

Reviewer comments
