# Supplementary material for: Precursor types predict the stability of neuronal branches
Source: J Cell Sci. 2021 Dec 6;134(23):jcs258983. doi: 10.1242/jcs.258983 (PMC8714070; doi:10.1242/jcs.258983)
Supplement: Supplementary information [file joces-134-258983-s1.pdf]

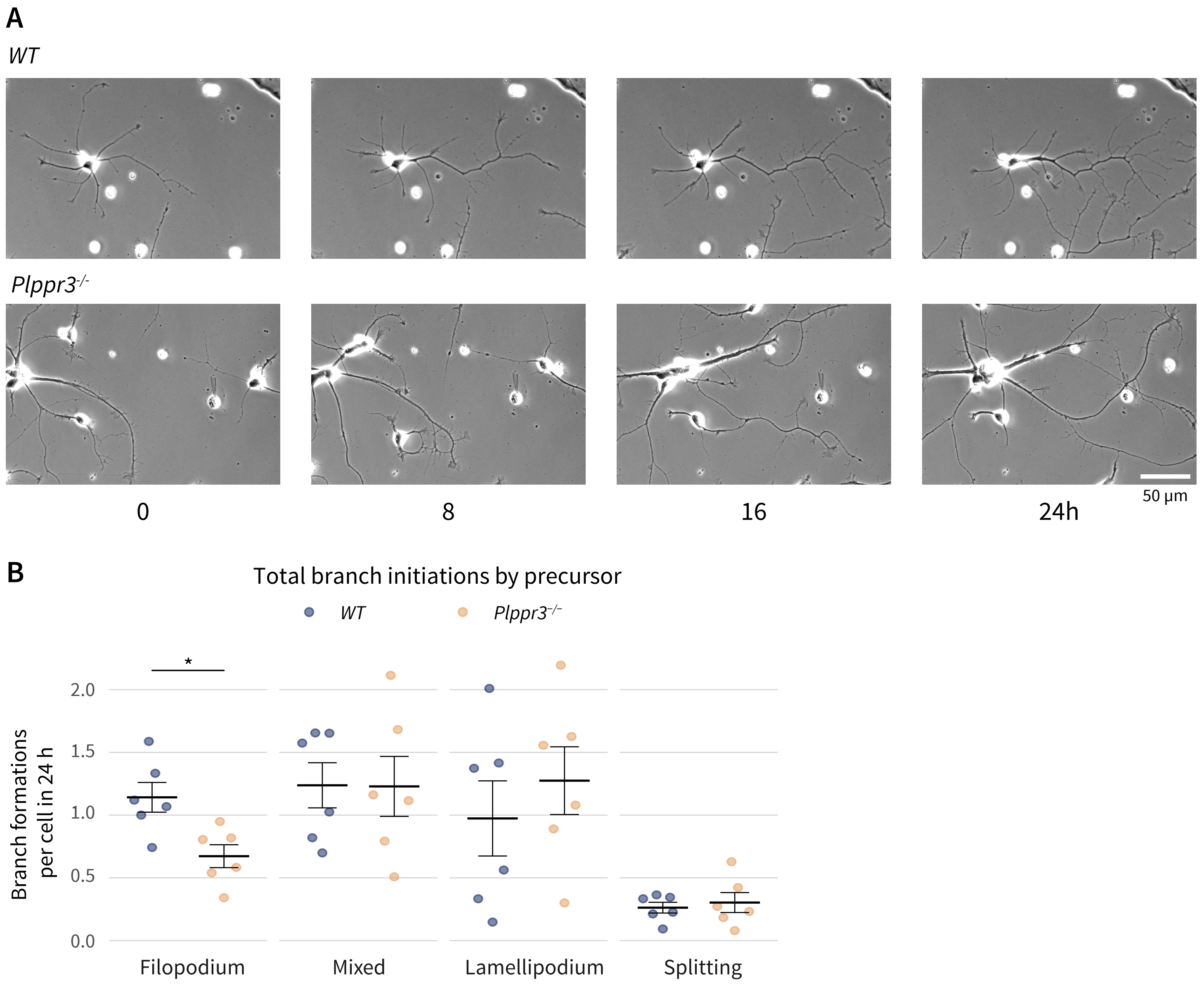

Fig. S1. Branching in *wildtype* versus *Plppr3*$^{-/-}$ neurons.
(A) Timeseries of Movie S1 of *WT* and *Plppr3*$^{-/-}$ mouse hippocampal neurons. Note the higher increased dynamics of branches in *Plppr3*$^{-/-}$. (B) Total branch formations per cell over 24 h, originating from different precursor types and genotype. *Plppr3*$^{-/-}$ neurons initiate fewer branches from filopodia. Error bars in (B) indicate s.e.m., data were tested with one-way ANOVA and post-hoc Welch's t-test with Holm correction for multiple testing. * $p < 0.05$.

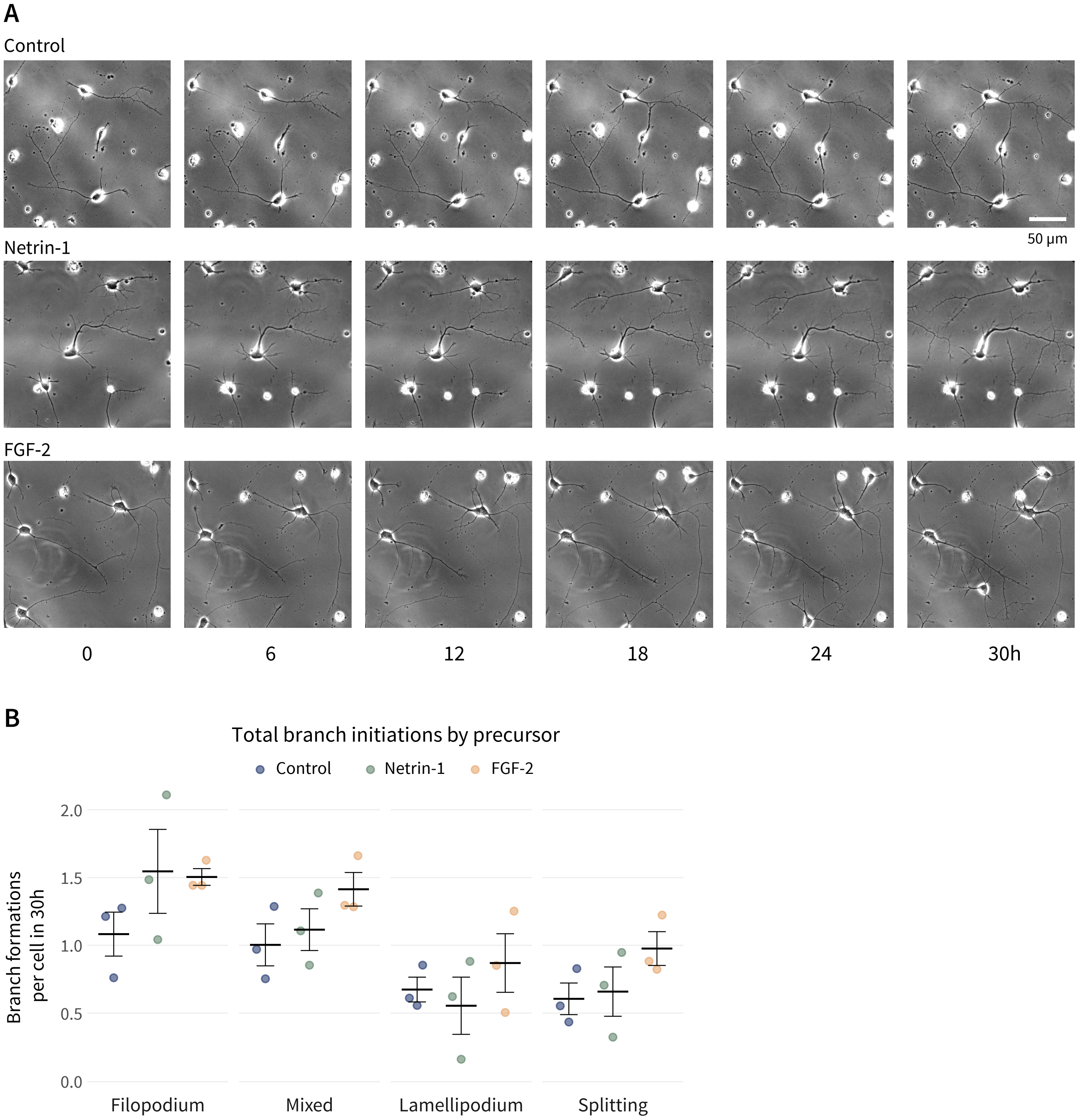

Fig. S2. Branching inducing treatments Netrin-1 and FGF-2
(A) Timeseries of Movie S2 of control, Netrin-1- and FGF-2 treated mouse hippocampal neurons. Note the increased branching from filopodia after Netrin-1, and the increased branch dynamics after FGF-2 treatment (B) Total branch formations per cell over 30 h, originating from different precursor types segregated by treatments. Netrin-1 treated neurons trend to initiate more branches from filopodia, FGF-2 treated neurons trend to initiate more branches from all precursors. While these effects are in line with published evidence on Netrin-1 and FGF-2 (Dent, 2004), they were not statistically detectable at three experiments and should be verified in future confirmatory studies. Error bars in (B) indicate s.e.m., data were tested with one-way ANOVA and post-hoc Welch's t-test with Holm correction for multiple testing.

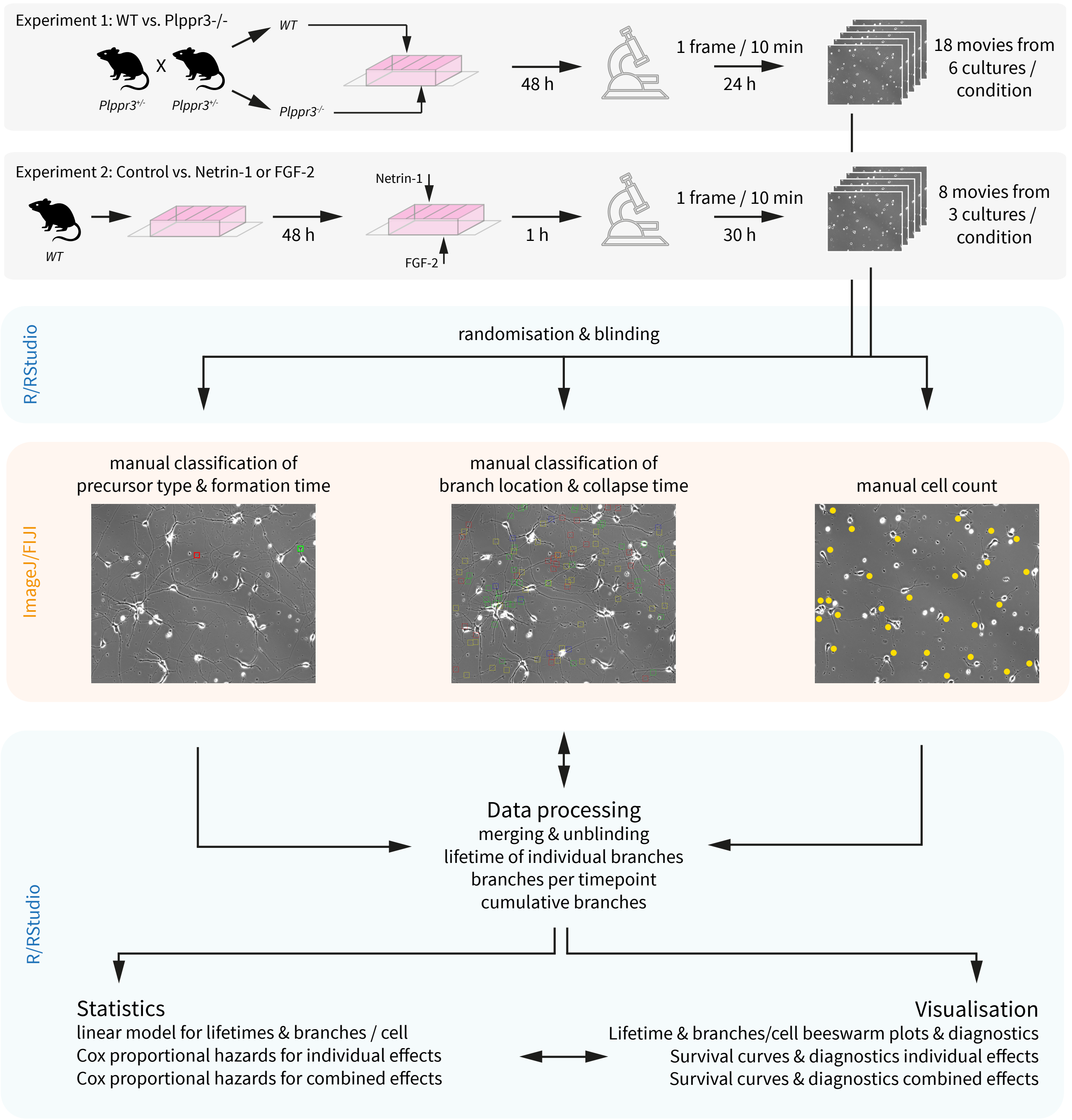

Fig. S3. Workflow of experiments and analysis

Experiment 1: *Plppr3*$^{-/-}$ and *WT* hippocampal neuron cultures were prepared from littermates of heterozygous *Plppr3*$^{+/-}$ mice and grown on poly-ornithine & laminin-coated 4-well chamber slides for 48 h. Experiment 2: *WT* hippocampal neuron cultures were grown on poly-ornithine & laminin-coated 4-well chamber slides for 48 h before treating wells with 100 ng/ml Netrin-1, 20 ng/ml FGF-2, or an equal volume of medium for 1 hour before acquiring images. Branching was imaged using phase contrast (Ph2) light microscopy for 24 h (Experiment 1) or 30 h (Experiment 2) with an interval of 10 min. Before manual branch classification in FIJI, each experiment was randomised and blinded to reduce bias in quantification. The analysis consisted of a three-step process, first classifying formation time and precursor type followed by collapse time and neurite type of individual branches. Finally, cells per movie were counted to normalise differences in density between cultures. All subsequent steps of the analysis were performed using R/RStudio and are fully documented at https://github.com/jo-fuchs/Branch-Lifetime-PRG2.

Table S1. Key resources

Reagent or Resource	Source	Identifier
**Deposited data**		
Individual branch classifications and lifetimes (Individual_branches.csv)	This paper	https://github.com/jo-fuchs/Branch-Lifetime-PRG2
Cells number and genotypes per movie in analysis (Cells.csv)	This paper	https://github.com/jo-fuchs/Branch-Lifetime-PRG2
**Experimental Models: Organisms/Strains**		
C57Bl/6 NCrl *Plppr3*$^{-/-}$ mice	Brosig et al., 2019	-
C57Bl/6 NCrl mice	Charles River	Cat# CRL:027 RRID: IMSR_CRL:027)
**Chemicals**		
Netrin-1	R&D Systems	Cat#: 1109-N1
FGF-2 (FGF-basic)	PeproTech	Cat#: AF-100-18B
**Software and Algorithms**		
Full analysis scripts in R	This paper	https://github.com/jo-fuchs/Branch-Lifetime-PRG2
FIJI	Schindelin et al., 2012	https://imagej.net/Fiji
R	R Core Team, 2020	http://www.r-project.org/index.html

Journal of Cell Science • Supplementary information

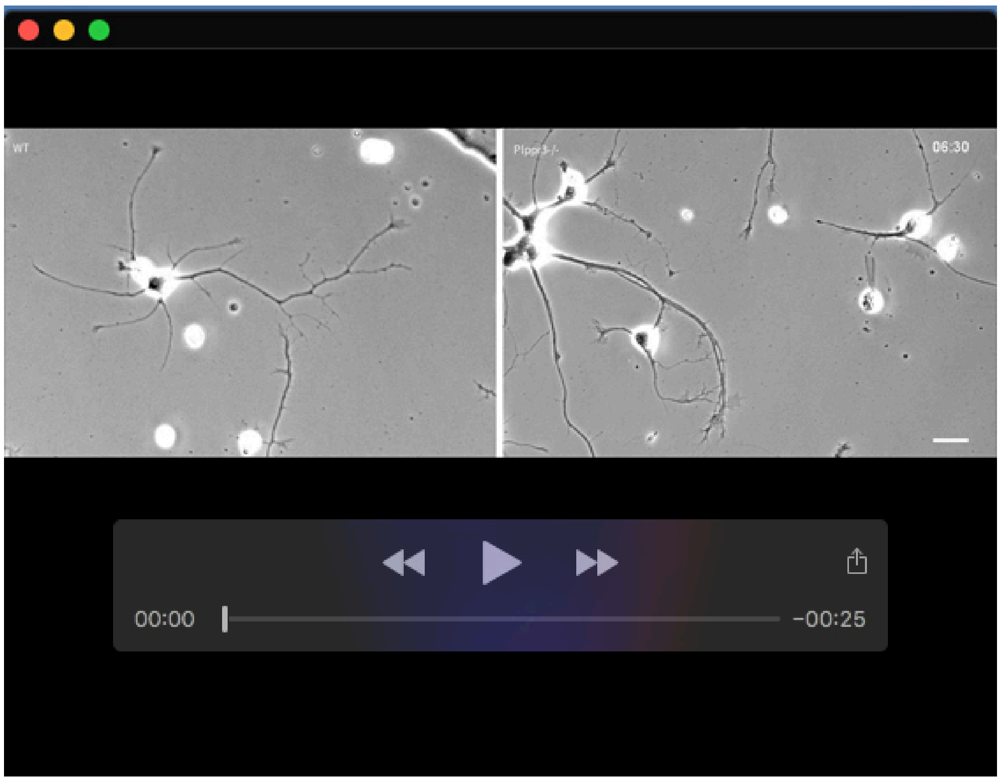

Movie 1. Branching in primary wildtype versus *Plppr3*$^{-/-}$ mouse hippocampal neurons. 24-hour phase-contrast time-lapse movie with 10 min intervals starting at day in vitro 2. Scale: 20 µm.

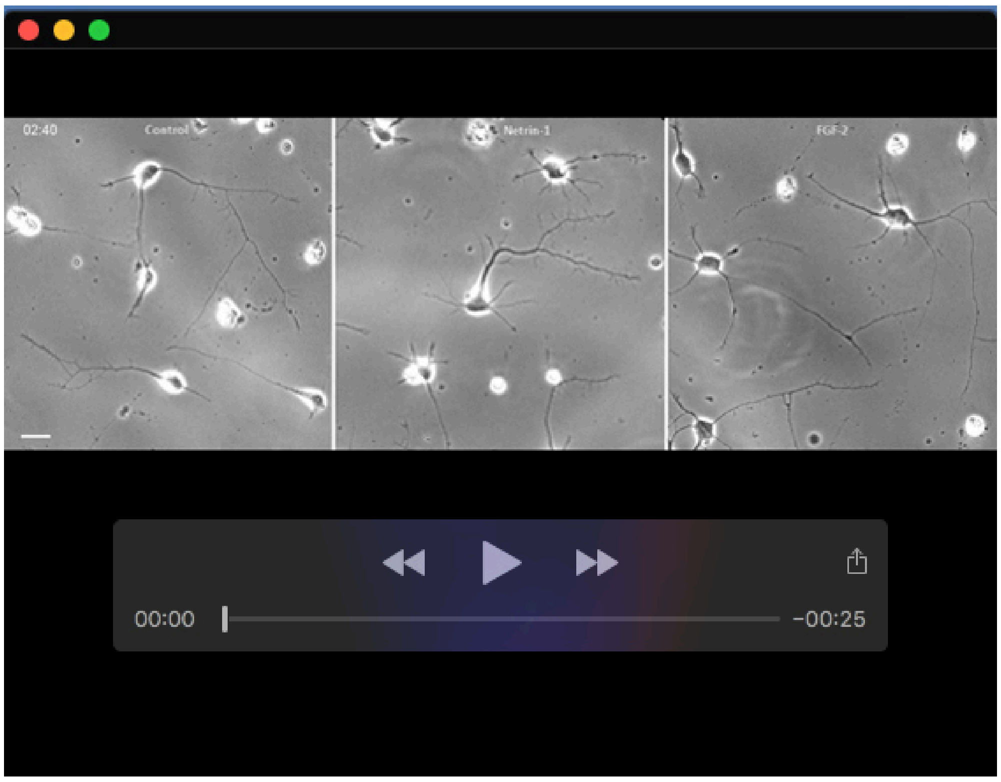

Movie 2. Branching in primary wildtype mouse hippocampal neurons after Netrin-1 or FGF-2 stimulation. 30-hour phase-contrast time-lapse movie with 10 min intervals starting at day in vitro 2. Treatments were applied one hour before imaging. Netrin-1: 100 ng/ml, FGF-2: 20 ng/ml. Scale: 20 µm.

Journal of Cell Science • Supplementary information
