# Supplementary material for: Precursor types predict the stability of neuronal branches
Source: J Cell Sci. 2021 Dec 6;134(23):jcs258983. doi: 10.1242/jcs.258983 (PMC8714070; doi:10.1242/jcs.258983)
Supplement: Reviewer comments [file joces-134-258983_review_history.pdf]

Precursor types predict the stability of neuronal branches

Joachim Fuchs and Britta J. Eickholt
DOI: 10.1242/jcs.258983

Editor: Giampietro Schiavo

Review timeline

Original submission:	2 June 2021
Editorial decision:	12 July 2021
First revision received:	22 October 2021
Accepted:	3 November 2021

Original submission

First decision letter

MS ID#: JOCES/2021/258983

MS TITLE: Precursor types predict the stability of neuronal branches

AUTHORS: Joachim Fuchs and Britta J Eickholt
ARTICLE TYPE: Short Report

We have now reached a decision on the above manuscript.

To see the reviewers' reports and a copy of this decision letter, please go to: https://submit-jcs.biologists.org and click on the 'Manuscripts with Decisions' queue in the Author Area. (Corresponding author only has access to reviews.)

As you will see, the reviewers gave favourable reports but raised some critical points that will require amendments to your manuscript. I hope that you will be able to address at least some of these points since they will boost the impact of your manuscript.

We are aware that you may be experiencing disruption to the normal running of your lab that makes experimental revisions challenging. If it would be helpful, we encourage you to contact us to discuss your revision in greater detail. Please send us a point-by-point response indicating where you are able to address concerns raised (either experimentally or by changes to the text) and where you will not be able to do so within the normal timeframe of a revision. We will then provide further guidance. Please also note that we are happy to extend revision timeframes as necessary.

Please ensure that you clearly highlight all changes made in the revised manuscript. Please avoid using 'Tracked changes' in Word files as these are lost in PDF conversion.

I should be grateful if you would also provide a point-by-point response detailing how you have dealt with the points raised by the reviewers in the 'Response to Reviewers' box. Please attend to all of the reviewers' comments. If you do not agree with any of their criticisms or suggestions please explain clearly why this is so.

© 2021. Published by The Company of Biologists under the terms of the Creative Commons Attribution License (https://creativecommons.org/licenses/by/4.0/).

Reviewer 1

Advance summary and potential significance to field

Fuchs et al report that the loss of neurite branching observed in PLPPR3 knock-out mice is due to a loss of filopodia generation and that generally branch stability is mostly determined by their precursor type:
Filopodia have a higher likelihood to form persisiting branches than lamella or split growth cones. Using multifactorial statistics, the authors exclude a direct link between PLPPR3 and branch stability but argue that the overall loss of branches (and hence their stability) is due to the reduction of filopodial branch progenitors.

For this study the authors imaged dissociated hippocampal neurons at DIV2 using phase contrast imaging and manually annotate the branch phenotypes, locations and timepoints. The overall conclusion of this paper is plausible and the methodological point made by the authors, that multifactorial analysis can reduce the number of interdependent but independent connections in biological pathways, is important in light of the ever-growing number of observations published. However, the biological implications of this paper are minimal. To emphasize the importance of multifactorial analyses it would be of interest to see such analysis applied to several pathways (in addition to PLPPR3), for instance general perturbations (using drugs) such as remodeling actin dynamics - jasplakinolide and latrunculin and microtubule dynamics - by for example taxol and nocodazole. In addition, I truly appreciate the point the authors make about the statistics. However the data is very limited - it is basically annotated 18 timelapse movies and analysed it 3 times in different groups). I'd suggest the authors work out either side better (two major comments): show more processes that have indirect interdependent variables (see suggestion above about cytoskeleton drugs) or explain more why filopodia are stable and lamella not.

Comments for the author

More specific comments
1. The dataset underlying this study is limited. While there are sufficient number of cells and individual neuron cultures, all analyses spread over Fig 1-3 are the same data points (18 timelapse movies), split into different groups and analyses.

2. The distinction between axonal and dendritic branches is purely based on morphology, without validation by marker proteins, etc. In addition, there is a group of branches that fall into an ‘unclear’ group of which the cell bodies are out of the FOV. This classification is further ambiguous as the text refers to ‘immature dendrites’ as neurites, making it hard to follow which branches are analysed.

3. The mechanisms behind the stabilization of filopodial branches are briefly discussed yet there is no testing of the hypothesis or the underlying pathways. It would be beneficial to show factors that do independently regulate branching stabilization to contrast PLPPR3’s indirect contribution.

4. The mechanisms behind lamellipodia vs filopodia generation for branching are also not studied in this report beyond the role of PLPPR3. It would be interesting to induce more of either kind of actin structures to test if the conclusion that filopodia are always more stabilized is true.

5. Plotting branch lifetime as time of formation vs collapse (e.g. Fig1B) is confusing. Do the authors expect differences in the lifetimes over the time of image acquisition? A horizontal bar graph with sorted life times would be more informative for the reader. Same for Fig 3A.

6. Counting the last timeframe as a ‘collapse’, as shown in this graph, is not correct as many of these branches will persist beyond the imaging window, as the authors admit. Restricting this analysis to branches with defined formation and collapse timepoints would give a better impression of their lifetime.
In addition, the number of branches exceeding a timeframe considered temporary could be shown.

© 2021. Published by The Company of Biologists under the terms of the Creative Commons Attribution License (https://creativecommons.org/licenses/by/4.0/).

7. The figure legend of Figure 2 mentions both WT and KO cells, is this figure pooling genotypes in this analysis or is this only done on WT cells? Similarly, is this analysis in 1 D and E performed on pooled genotypes?

8. The authors mention actin waves, temporary actin dynamics that move long developing neurites. Not all of these dynamics are associated with branching activity, how is this distinguished in your data set?

9. Figure S2 should be part of the main figure. This graph clearly shows the contribution of each variable in your model and is a main result explaining the validity of Fig 3D.

10. The naming of branches and their ‘progenitors’ is inconsistent throughout the manuscript.

11. I suspect the y axis labels in Fig 3A/B lack a time unit. Are those branches / cell / hour? Similar in Fig 3C, is there only 1 filopodium made per cell per 24h? Are the time units correct?

12. The “standard method” used to determine the effect of the PLPPR3-/- genotype on branch stability should be clarified (why did this method produce significant differences)

13. Why was the effect determined to not be directly linked to PLPPR3 and instead due to inducing filopodia?

14. “The stabilizing effect in developing axons is likely influenced by differences in orientation and post-translational modifications of microtubules/MAPs..” Is there any direct evidence for this?

15. Is it known what “actin precursors” are present in all of these branch types from a mechanistic standpoint? Bifurcations, collateral, filopodia vs. lamellipodia 16. Data should be reorganized to first explain the differences of branches from axons and neurites and then transition to the PLPPR3 data

Reviewer 2

Advance summary and potential significance to field

This submission presents evidence addressing the relationship between the type of actin filament based structure that gives rise to an axon collateral branch and the stability of the branch, as well as differences in branch stability between immature dendrites and axons and non-collateral based forms of axon branch formation. The information is used to elucidate aspects of KO phenotype that affects branches. A type of quantitative analysis, termed multifactorial causal analysis, not common in cell biology is used to extract information from the data sets. This is a very well presented and compelling manuscript and makes important novel observations about a fundamental aspect of branch stability. Constructive suggestions for the author’s consideration are presented below.

Comments for the author

Results
Lines 84-91. Although the argument provided that the effects of Plpp3-/- on branch stability is due to a decrease in the number of filopodia formed by the -/- axons relative to WT is reasonable, an analysis of filopodial dynamics would be insightful. Might Plpp3-/- also be affecting filopodial stability? Furthermore, in returning to Borsig et al 2019, filopodia were assessed morphometrically but not through live imaging (lest I mist it). Thus, changes in the number/density of filopodia along axons could have arisen from differences in the rate of formation of filopodia or the stability of filopodia after forming, or both. Inclusion or an analysis of filopodia rate of formation and stability could provide insights into the major issue. Similarly, in WT do collateral branches arise preferentially from longer lived filopodia? More stable filopodia might reflect differences in the cytoskeleton prior to the formation of a branch from the filopodium that are then maintained in the growing branch.

© 2021. Published by The Company of Biologists under the terms of the Creative Commons Attribution License (https://creativecommons.org/licenses/by/4.0/).

The section in Statistical analysis states that Welch t-tests were used but does not address whether the data sets the parametric test was applied to were determined to be normal.
As studies of branching usually do not clump immature dendrites and axons in the same data sets (if ever) but differentiate between the two types of processes, it is not clear to me why what is referred to as “location” (i.e., immature dendrites vs. axons) is considered a variable of value in the multifactorial analysis. A rationale for the inclusion of “location” in the multifactorial analysis, as it would apply other studies on branching, would benefit the reader.

Introduction, Discussion and Presentation
As the importance of considering multifactorial causal analysis in cell biological studies is a major claim/conclusion of the work, I would suggest the presentation would greatly benefit for a much clearer exposition of the concept and method (as indeed most of us cell biologist are likely not knowledgeable about it, this was my first introduction to it). I would suggest two major revisions; (1) The introduction would greatly benefit from a clear paragraph presenting the analytical approach thereby priming the reader for what is to follow. (2) In the results, specifics about the analysis be presented to assist the reader in following how the information is extracted from the data sets using the analysis. (3) One aspect of the presentation that would benefit from revision is an early introduction to causal graphs. When Fig 1F is referenced, the reader is left to wonder what the significance of panel F may be. Indeed, it is only referenced as a cluster 1D-F and no specific attention is drawn to it in the text and the legend only provides a statement of fact about the panel; that it is a causal graph, but as a reader I had no idea what that meant or how it contributed. (4) a specific example: Line 105. Here again a clearer presentation of causal graph theory would greatly assist the reader. What is a "collider"? (4) Line 48 is the only mention of “hazard ratio” and line 236 the only mention of “Cox proportional hazards models”, and Supp Fig 2 is referenced without any clear discussion/presentation. Here again, a naïve reader not familiar with the method is at a complete loss (I certainly was). In conclusion, the authors are urged to provide a primer for the readers regarding the analytic method being applied. It would really help a reader if they were “taken by the hand” and walked through the method step by step with rationales and justifications provided along the way.

Lines 93-94. I do not follow what the authors mean when they write that " We showed that PLPPR3-loss decreases branch stability and reduces the numbers of the most efficient precursor (filopodia), preferentially on axons, which themselves stabilise branches in two distinct ways". In what two distinct ways do filopodia stabilize branches? (also, minor spelling error; stabilise should read stabilize, and I found 4 occurrences of the misspelling).

Line 115-116. "We identified that filopodia are not the most abundant precursor, but the most efficient". Lest I am misreading Fig 3A, filopodia account for approximately 50% of collateral branches, and the majority of the rest of the branching is through mixed filopodia-lamellipodia structures. The latter could also be argued to be argue to be mostly filopodial as the mixed are described as filopodia that then transition to having lamellipodial structures. Thus, it is not clear to me how the claim that filopodia are not the most abundant precursor.

The discussion of the possible cell biological underpinnings of the differences in branch stability between filopodia and lamellipodia formed branches may benefit from consideration of studies in embryonic sensory neurons, where it is my understanding the majority of branches arises from filopodia precursors. The base of filopodia along the axon contains septin-7 and drebrin, two proteins that assist in targeting microtubules into filopodia and early branches (Curr Biol. 2012 Jun 19;22(12):1109-15; Dev Neurobiol. 2016 Oct;76(10):1092-110) and these proteins are retained at the base of branches or are present shaft as they elongate. To my understanding neither septin-7 nor drebrin are known to mediate functions in lamellipodial actin networks. Thus, proteins such as septin-7 and drebrin may contribute to the greater stability of branches formed from filopodia. It would be interesting is these proteins were not found associated with the bases or shafts of branches that have arisen from lamellipodia, but at this point it’s a discussion item.
Minor suggestion: the term "location" to differentiate whether a branch formed from an axon or an immature dendrite in the text and Fig 1F may not be the best choice of terminology as what is being differentiated are two different types of neuronal processes. Personally, upon reading about "location" I assumed the authors might refer to location along the axon shaft (e.g., closer or further from the cell body). Thus, I would suggest revising the terminology and switching from "location" to "type of neuronal process" or more directly "Axon vrs Immature Dendrite".

© 2021. Published by The Company of Biologists under the terms of the Creative Commons Attribution License (https://creativecommons.org/licenses/by/4.0/).

Reviewer 3

Advance summary and potential significance to field

In this short article, Fuchs and Eickholt, use detailed analysis of branch formation and survival from different structures (filopodia, lamellipodia, growth cones), neuronal compartments (neurites, axons) and genotypes (wt vs. plppr3) in order to demonstrate in a very elegant way the factors associated to branch stability. By using survival curves and causal diagrams, they develop a method that can be used to test the influence of multiple variables in the final outcome in this case branch stability. By doing so, they also demonstrate that PLPPR3 modify branch numbers by indirectly regulating branch stability influencing branch precursor type. The analysis performed are very detailed and methods used for analysis allows the authors to establish several important conclusions which opens avenues for mechanistic investigation of the molecular events associated to each structure, including the influence of different cytoskeletal configuration and cross linking proteins.

Comments for the author

A couple of minor issues can a strength this work, which are indicated below.
-It will be interesting to better define the mixed precursor branches, and if after closer inspection and analysis of their behavior they can be assigned as finally originated from filopodia or lamellipodia. If not possible, then it will be important to discuss if the mechanisms associated to the formation of these mixed precursor branches is qualitatively different from the ones originating from filopodia or lamellipodia. Looking at survival in Fig.2C it seems that they behave more like a lamellipodia-associated branch.
-It will be important to extend the discussion about multifactorial statistical analysis, in terms of the requirements for this type of analysis. As the author state, this type of analysis can really advance the definition of relationships in multifactorial experiments, and the readers will benefit form some details about their implementation.

First revision

Author response to reviewers' comments

Reviewer #1

To emphasize the importance of multifactorial analyses it would be of interest to see such analysis applied to several pathways (in addition to PLPPR3), for instance general perturbations (using drugs) such as remodeling actin dynamics - jasplakinolide and latrunculin and microtubule dynamics - by for example taxol and nocodazole. In addition, I truly appreciate the point the authors make about the statistics. However the data is very limited - it is basically annotated 18 timelapse movies and analysed it 3 times in different groups).

I'd suggest the authors work out either side better (two major comments): show more processes that have indirect interdependent variables (see suggestion above about cytoskeleton drugs) or explain more why filopodia are stable and lamella not.

We thank the reviewer for these excellent suggestions. We strongly agree that an independent dataset strengthens both claims about biology and analysis strategy. We furthermore consider both questions raised by the reviewer extremely relevant to the presented results. Specifically, the question how precursor types could affect the stability of branches (which is executed long after the precursor types have been replaced by a branch) is an intriguing direct follow up for the main claim of this study that uncovers this connection. The molecular implementation likely involves precursor-type specific proteins (or organelles) with functions that have not yet been uncovered or connected to the process of mediating branch maintenance. We therefore believe that this question is more likely to be solved by efforts of multiple labs and goes beyond our first characterisation of this sequence of events.

© 2021. Published by The Company of Biologists under the terms of the Creative Commons Attribution License (https://creativecommons.org/licenses/by/4.0/).

To address this comment, firstly, we extended the discussion on putative molecular mechanisms (lines 164- 166, lines 168-170, and 174-177). Secondly, we provided an additional line of evidence to broaden the implications of our main finding. To this end, we utilised two external treatments to increase precursors, namely netrin-1 and FGF-2, and quantify the magnitude and type (direct or indirect) of effects on branch stability. This independent dataset is now included as Fig. 4 (lines 116-149, Fig. S2 and Movie S2).

We chose, not to adopt the proposed pharmacological perturbation of F-actin or microtubule stability, as previous studies suggest that these treatments affect several stages of the branching program as well as general neuron development. Altering microtubule dynamics using nocodazole or taxol have been found to both decrease the number and length of branches and the axon in cortical neurons (Dent and Kalil, 2001). Nocodazole predominantly affects elongation and stabilisation of developing dendrites and minor processes on the axon (Witte et al., 2008), nocodazole and taxol both decrease the initiation (the invasion of microtubules into filopodia) by 60% without altering growth-retraction dynamics of branches drastically (Gallo and Letourneau, 1999). Nocodazole (and the disruption of F-actin dynamics by cytochalasin D) have been suggested to suppress specifically actin waves (Flynn et al., 2009), and while Latrunculin B appears to specifically decrease branch formation, jasplakinolide decreases growth in addition to branch formation (Willige et al., 2019). With these manifold effects on precursor initiation, precursor to branch transition, branch elongation, and branch stabilization, we were unsure how to specifically differentiate effects of these treatments on branch stability. We hope the alternative strategy to specifically increase precursor types convinces the reviewer as an appropriate extension of our results demonstrating that precursor types affect branch stability.

1. The dataset underlying this study is limited. While there are sufficient number of cells and individual neuron cultures, all analyses spread over Fig 1-3 are the same data points (18 timelapse movies), split into different groups and analyses.

We agree that the focus on one dataset is a limitation of the study. We want to emphasise, however, that we have extracted extensive amounts of information (four descriptive and predictive variables of 4482 branching events) from 532 wildtype and 466 Plppr3$^{-/-}$ neurons from (in total) 36 timelapse movies, to describe neuronal branching in a detail that has, to our knowledge, not been presented previously. We furthermore believe, that spreading the presentation of the analyses over multiple figures was necessary to present all implications in a truthful as well as accessible way.

We hope we have addressed this concern by including a new dataset (including 5459 branching events in a longer observational time window (30 hours) from (in total) 576 cells of three cultures treated with different branch inducers) that provides consilient evidence to the inferences drawn from the original dataset.

2. The distinction between axonal and dendritic branches is purely based on morphology, without validation by marker proteins, etc. In addition, there is a group of branches that fall into an 'unclear' group, of which the cell bodies are out of the FOV. This classification is further ambiguous as the text refers to 'immature dendrites' as neurites, making it hard to follow which branches are analysed.

We agree that the study would benefit from a more detailed description of neurite types. We chose to classify purely by morphology, as many live-cell axon makers (e.g., Tau or TRIM46) have described overexpression effects on branching or microtubule stability (Tymanskyj and Ma, 2019; Van Beuningen et al., 2015; Yu et al., 2008), thereby potentially modifying the process we study. After establishing that marker proteins or long-term fluorescent imaging do not affect this specific readout, this approach could, however, allow for a more precise characterisation of branch programs on axons and dendrites.

The 'unclear' category was included to extract as much information as possible from all branches in each movie. While it is unclear whether these processes are axon or immature

© 2021. Published by The Company of Biologists under the terms of the Creative Commons Attribution License (https://creativecommons.org/licenses/by/4.0/).

dendrite, they still should contribute to the lifetime estimation of branches from different precursor types or genotypes. Similar reasoning applies to 'mixed precursor' associated branches. We are unsure whether this 4th category has completely independent or shared biological mechanisms with filopodia or lamellipodia induced branches. Nevertheless, they contribute to the overall stability of axonal or Plppr3$^{-/-}$ branches and excluding them from an analysis could potentially bias the estimation of total effects. In the interest of clarity, we have changed the name from 'neurites' to 'immature dendrites', as well as 'location' to 'neurite type'.

3. The mechanisms behind the stabilization of filopodial branches are briefly discussed yet there is no testing of the hypothesis or the underlying pathways. It would be beneficial to show factors that do independently regulate branching stabilization to contrast PLPPR3's indirect contribution.

This study uncovered the existence of a physiological mechanism in which neuronal branch stability is pre- determined at the timepoint of formation, suggesting the existence for distinct molecular mechanism between branches associated with distinct precursors. We agree that the molecular implementation of how filopodia set the stage for stable branches is an interesting question. To facilitate and guide such studies, we have extensively discussed potential starting points for mechanistic studies and have extended this discussion (according to suggestions from Reviewer 2).

With the new dataset, we have provided consilient evidence that precursor types predict branch stability: netrin-1 indirectly increases branch stability via the precursor type - precisely reversing the Plppr3$^{-/-}$ effect. Furthermore, and even more intriguingly, FGF-2 appears to decrease branch stability by mechanisms that can't be explained with alterations of precursor type composition, which further reinforces the methodological strengths of utilising multifactorial analyses.

4. The mechanisms behind lamellipodia vs filopodia generation for branching are also not studied in this report beyond the role of PLPPR3. It would be interesting to induce more of either kind of actin structures to test if the conclusion that filopodia are always more stabilized is true.

We want to thank the reviewer for this insightful comment. We included a new dataset using netrin-1 and FGF-2 to alter precursor numbers and study their effect on branch stability (Fig. 4, supplementary Fig. S2, Movie S2 and lines 116-149). These experiments support the original dataset on wild type versus Plppr3$^{-/-}$ neurons.

5. Plotting branch lifetime as time of formation vs collapse (e.g. Fig1B) is confusing. Do the authors expect differences in the lifetimes over the time of image acquisition? A horizontal bar graph with sorted life times would be more informative for the reader. Same for Fig 3A.

Fig. 1B (now 1A), 1D, 2B and 4C illustrate the strong censoring in the datasets to motivate the choice of survival analysis rather than directly comparing lifetimes over time. We have not quantified differences in the lifetimes over the time of image acquisition but consider it an interesting question. However, the time window in these datasets is unfortunately too short for comparisons of raw lifetimes.

A histogram of lifetimes provides (to our understanding) the same information as the previous Fig. 1A, which is a problematic representation of lifetime data. If 'horizontal bar graph' refers to a graph of lifetimes over time, the same criticisms apply. Later timepoints have shorter remaining observational time windows; therefore, observed lifetimes would appear shorter than they are in reality. Response figure 1 illustrates how in both datasets, an unbiased measure of the raw lifetime is not possible regardless of restricting a quantification on complete observations or observing lifetimes for longer (new 30h dataset). The observable lifetime of a branch always depends on when it formed in the movie. Further discussion to this point is provided in the answer to comment 6.

© 2021. Published by The Company of Biologists under the terms of the Creative Commons Attribution License (https://creativecommons.org/licenses/by/4.0/).

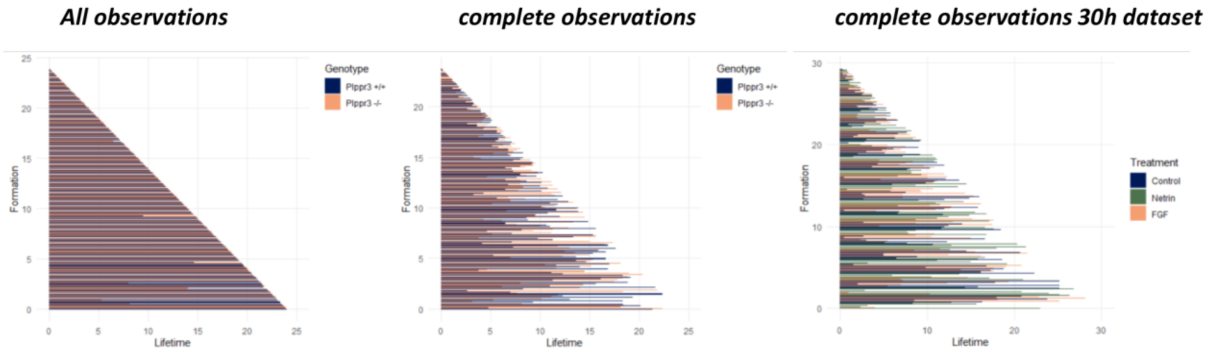

Response figure 1: Lifetime by timepoint of formation in both datasets. Note how even complete observations of the lifetime show a clear dependence on the timepoint of formation. Any analysis of raw lifetimes would be biased towards shorter lifetimes.

6. Counting the last timeframe as a 'collapse', as shown in this graph, is not correct as many of these branches will persist beyond the imaging window, as the authors admit. Restricting this analysis to branches with defined formation and collapse timepoints would give a better impression of their lifetime. In addition, the number of branches exceeding a timeframe considered temporary could be shown.

We agree that counting the last timeframe as a collapse would create a drastically biased estimate of a raw lifetime measure as presented in Fig. 1A. Indeed, we discussed this issue in the paper and used Fig. 1A and B to illustrate why such an analysis would be incorrect. This then motivated our choice for exploiting the less familiar (but very widely used) 'survival analysis'. The results in Fig. 1A were therefore not supposed to be interpreted beyond this explanatory purpose, as they misrepresent the underlying data. To avoid further confusion, we have modified our introduction and motivation for 'survival analysis' (lines 39-46) and have removed panel A from Fig. 1.

Restricting the analysis to complete observations, as suggested by the reviewer, would, however, also introduce bias in the estimation of lifetimes. The chance for complete observations depends on the length of observation, as well as on the timepoint of formation (see Response figure 1). Especially for long-lived branches (such as those originating from filopodia), the restriction to analyse complete observations would severely underestimate the true lifetime and thereby mask effects. Similarly, dichotomising the continuous lifetime data by comparing ratios of 'stable' branches to 'temporary' branches ignores information about how long a branch has been stable. A branch forming 20 minutes after the timelapse started that is still present after 24 hours should influence the analysis differently from a branch forming at 10 hours or 23 hours. A 'temporary' time window would introduce further subjectivity and only inefficiently utilise the information in the <u>observed</u> lifetime of non-collapsed branching.

To represent information truthfully and efficiently in such data, survival analysis methodologies treat branches that persist beyond 24 hours as censored datapoints, which influence the estimation of treatment effects differently than branches with a completely observed lifetime. To this end, survival analyses do not estimate exact lifetimes, but instead a risk for collapsing (in case of Cox proportional hazard analysis the reported hazard ratio) which has, unfortunately, less intuitive interpretations. Survival analyses, however, include all observations and efficiently extract relevant data while correcting for potential bias through censoring. To further reduce bias, we introduced weights for observations based on how close they formed to the end of the movie $({}^{1}/_{t_{end}-t_{formation}})$. For further information why and how survival analysis strategies treat censored data more efficiently and with less bias than splitting a dataset into complete and censored cases we refer to Leung et al., 1997.

© 2021. Published by The Company of Biologists under the terms of the Creative Commons Attribution License (https://creativecommons.org/licenses/by/4.0/).

7. The figure legend of Figure 2 mentions both WT and KO cells, is this figure pooling genotypes in this analysis or is this only done on WT cells? Similarly, is this analysis in 1 D and E performed on pooled genotypes?

In figures 1 and 2, we specifically included both genotypes. We argue that the effects of precursor type and neurite type appear in both genotypes and are important to explain the total effects of all factors in the full dataset including both genotypes (Fig. 3). In this way, we hope to facilitate the comparison of the effects reported from figures 1 and 2 to the effects of the full model in Fig. 3D.

For reference we included a comparison of Hazard ratios for only wild type (WT) to the complete dataset (Full) Hazard ratios (Table 1). Note that for none of the factors statistical detectability is affected and that the confidence intervals are largely overlapping. The interpretation of the effects in the dataset does not change whether wild type or both genotypes are analysed.

Table 1: Hazard ratios of analyses presented in Figures 1 and 2 compared to hazard ratios of only wild type dataset

Branch precursor	***Hazard ratio (Full)***	***Hazard ratio (WT)***
Filopodium	reference	reference
Mixed	2.6 (2.1-3.3), p<0.001	2.3 (1.6-3.2), p<0.001
Lamellipodium	4.6 (3.6-5.7), p<0.001	4.0 (2.9-5.5), p<0.001
Splitting	5.0 (3.8-6.6), p<0.001	4.8 (3.1-7.2), p<0.001
Neurite type		
Axon	reference	reference
Dendrite	5.6 (4.6-6.7), p<0.001	5.2 (4.6-5.9), p<0.001
Unclear	2.1 (1.6-2.8), p<0.001	1.9 (1.6-2.3), p<0.001

8. The authors mention actin waves, temporary actin dynamics that move long developing neurites. Not all of these dynamics are associated with branching activity, how is this distinguished in your data set?

The presented datasets focus on precursor events that form a branch, which are indeed a subset of all precursor events. Similar to actin waves, only a subset of filopodia initiates a branch. Therefore, we cannot (and do not) draw inferences about how many precursors initiate a branch. We only describe branch stability and its relation to the precursor it started from. We do not present data regarding branch initiation rates from precursors, nor do we present evidence regarding precursor stability; every readout presented refers to branches.

9. Figure S2 should be part of the main figure. This graph clearly shows the contribution of each variable in your model and is a main result explaining the validity of Fig 3D.

We agree and have moved the forest plot as a new panel in Fig. 3. We have also added the corresponding forest plots of the new dataset to the new Fig. 4. We want to note, however, that other Hazard ratios than those for 'Genotype' or 'Treatment' should be interpreted with care. The presented analyses were designed to address the question if Plppr3-genotype or treatment-effects are acting via precursor types only or via additional pathways. Hazard ratios of covariates might be confounded and depend on the exact structure of the statistical model (Westreich and Greenland, 2013).

© 2021. Published by The Company of Biologists under the terms of the Creative Commons Attribution License (https://creativecommons.org/licenses/by/4.0/).

10. The naming of branches and their 'progenitors' is inconsistent throughout the manuscript.

We thank the reviewer for this criticism. In an attempt to further clarify the description, we have adjusted the figures to explicitly state 'branch lifetime' and 'precursor type' (as was originally only specified only in the figure legends) and replaced 'actin precursor' with the more precise 'F-actin based branch precursor' throughout the manuscript. We hope these were the inconsistencies the reviewer was referring to.

11. I suspect the y axis labels in Fig 3A/B lack a time unit. Are those branches / cell / hour? Similar in Fig 3C, is there only 1 filopodium made per cell per 24h? Are the time units correct?

We thank the reviewer for pointing this out. In Fig. 3A & B, time is presented on the x-axis. For 3B the y- axis indicates the number of branches per cell (at a given time point on X). For 3A the y-axis represents the number of branch-forming events per cell at each given time point. Both figures show data binned into 2h to facilitate comparisons. This information has now been added to the figure legend.

Fig. 3C (now Fig S1B) presents <u>branch</u> initiations per cell in 24 hours from the respective precursors. The first comparison there does not present the total number of filopodia formed in 24 hours, but the number of branches forming from filopodia. Most filopodia did not transition into a branch and were therefore not included in this analysis (as explained in our answer to comment 8). We also expect the number of precursors to be considerably higher than the presented branch initiation events.

We have again verified the time units; they are indeed correct. The first dataset includes 2317 wild type branches from 576 cells in the observed 24 hours. Per cell this amounts to approximately four branch initiations which are distributed over the four precursors, resulting in the presented slightly more than one branch initiation per cell from filopodia. Due to the high turnover (short lifetime) of many branches, not all of those four initiated branches persist (as indicated in Fig. 3B). As a result, after 24 hours only about two branches were added in each cell (ca. 1.4 on axons, 0.4 on immature dendrites). Note that at the analysed developmental timepoint a considerable proportion of cells had very short neurites and did not yet polarise, such that many cells did not branch, while others formed a large amount.

12. The "standard method" used to determine the effect of the PLPPR3-/- genotype on branch stability should be clarified (why did this method produce significant differences)

Addressed in answer to comment 6. To avoid further confusion, we have excluded panel A from Fig. 1 and instead explained the choice for survival analysis methodology in more depth.

13. Why was the effect determined to not be directly linked to PLPPR3 and instead due to inducing filopodia?

The total effect of PLPPR3 on branch stability can be fully explained by its effect on modifying the amounts filopodia. In other words, filopodia-associated branches of Plppr3$^{-/-}$ neurons are as stable as filopodia- associated branches of wild type neurons and so is the case for all possible combinations of precursor and neurite type (Fig. 3E). Similarly, the effect of netrin-1 on branch stability can be fully explained by its effect on increasing the amounts of filopodia-associated branches (Fig. 4H & I). In contrast, the effect of FGF-2 on branch stability seems to be more complex (Fig. 4H & I).

14. "The stabilizing effect in developing axons is likely influenced by differences in orientation and post-translational modifications of microtubules/MAPs.." Is there any direct evidence for this?

We thank the reviewer for this critical question. We seem to have confused correlation for causation on this point. We have modified the corresponding section to more explicitly point towards microtubule associated proteins and clarified the role of orientation and modification in this respect (lines 57-61).

© 2021. Published by The Company of Biologists under the terms of the Creative Commons Attribution License (https://creativecommons.org/licenses/by/4.0/).

15. Is it known what “actin precursors” are present in all of these branch types from a mechanistic standpoint? Bifurcations, collateral, filopodia vs. lamellipodia

We thank the reviewer for this question, it has highlighted that ‘actin precursors’ might be a misleading phrase in this respect. We have, therefore, renamed the two occurrences to the more precise ‘actin-based branch precursors’ to avoid potential confusion by the reader.

To address this question: it is well established that growth cones contain filopodia and lamellipodial parts (Lowery and Van Vactor, 2009). In contrast, whether growth cone filopodia and lamellipodia are mechanistically and structurally similar to filopodia or lamellipodia on neurite shafts (which initiate the precursor events included in this study) is less clear.

This concerns also bifurcation, interstitial or terminal branching: Bifurcations arise from growth cone splitting or from collateral branching (Gibson and Ma, 2011), interstitial and terminal branches mainly form from collateral branches (Lewis et al., 2013). The extent to which collateral branches at the different anatomical locations are associated with the distinct precursor types has (to our knowledge) not been thoroughly quantified. Part of the reason might be, that filopodia and lamellipodia are surprisingly difficult to distinguish morphologically in three dimensions (Santos et al., 2020). Most studies focused on filopodia as the predominant collateral precursor, but some evidence also places lamellipodia as precursors for branches in vivo (Gascon et al., 2006).

It is, however, noteworthy that interstitial and terminal branching serve very different purposes and might therefore be implemented by complementary molecular mechanisms: terminal axon (and arguably most dendritic) branching increases the area of potential output (and input) for a neuron, while interstitial branching is required for innervating multiple brain regions or even proper targeting of the axon. In this respect, interstitial branching would benefit from committing, stable, branch initiations (as conceivable through filopodia) while dendritic (and terminal) branching even already has been described to utilise stochastic branching events (Castro et al., 2020; Snider et al., 2010) that could well be implemented by dynamic short-lived branch initiations from lamellipodia. While we find these ideas potentially interesting, we think they are currently too speculative to include them into the discussion of this present study.

16. Data should be reorganized to first explain the differences of branches from axons and neurites and then transition to the PLPPR3 data.

We thank the reviewer for the suggestion. We agree that this could be an alternative way of describing the data. For the main claim of this study as well as the methodological point for multifactorial analyses we believe, however, that the current organization works better.

Reviewer #2

1. Lines 84-91. Although the argument provided that the effects of Plpp3-/- on branch stability is due to a decrease in the number of filopodia formed by the -/- axons relative to WT is reasonable, an analysis of filopodial dynamics would be insightful. Might Plpp3-/- also be affecting filopodial stability? Furthermore, in returning to Borsig et al 2019, filopodia were assessed morphometrically but not through live imaging (lest I mist it). Thus, changes in the number/density of filopodia along axons could have arisen from differences in the rate of formation of filopodia or the stability of filopodia after forming, or both. Inclusion or an analysis of filopodia rate of formation and stability could provide insights into the major issue.

We thank the reviewer for this comment. Our data (from Brosig et al., 2019) suggest, that filopodia are PLPPR3-positive (amongst others Fig. S1 and S4 in Brosig et al., 2019) and that they form in higher proportions close to (pre-existing) PLPPR3 clusters (Fig. 6H, Brosig et al., 2019). This temporal precedence of PLPPR3 would suggest an initiation (gain-of-function) phenotype. But there is compelling evidence that PLPPR3-family members alter adhesive properties of cells (Liu et al., 2016; Tilve et al., 2020) and the precise mechanism for PLPPR-mediated filopodia phenotypes has not been fully elucidated. The suggested live-cell of

© 2021. Published by The Company of Biologists under the terms of the Creative Commons Attribution License (https://creativecommons.org/licenses/by/4.0/).

filopodia dynamics in wild type vs. Plppr3$^{-/-}$ could be a powerful way to address this question and is on our agenda. For this project, however, we do not believe that the exact mechanism by which PLPPR3 affects filopodia numbers alters the main conclusion that the type and mix of branch precursors determine branch stability. The present study merely uses the fact that PLPPR3 alters filopodia numbers to provide a link of precursor type composition to branch stability.

2. Similarly, in WT do collateral branches arise preferentially from longer lived filopodia? More stable filopodia might reflect differences in the cytoskeleton prior to the formation of a branch from the filopodium that are then maintained in the growing branch.

This is a very interesting idea. From preliminary inspection of our data, filopodia were usually more long- lived than other precursor types. Additionally, branches from filopodia seemed to elongate slower than those from mixed, lamellipodial or splitting precursors. It seems possible that part of the effect why filopodia are more efficient in recruiting branch-stabilising factors (to stabilise branches in the long-term) is mediated by the comparably longer lifetime of filopodia in our experimental system. In the same vein, long-lived filopodia could give rise to more persistent branches.

However, this hypothesis would be very difficult to test. Classifying branch lifetime and correlating branch lifetime with precursor lifetime (measuring both in the same movie) would require imaging at a time- resolution able to accurately resolve filopodia dynamics (10-30 sec intervals instead of 10 min), while still preserving the 24 hour (better longer) observational window. Manual analysis of such data would take extensive amounts of time even for multiple researchers (for comparison, single movies of the presented datasets here took on average 16 working hours to manually annotate). While we have tried several strategies to automatise the analysis, including intensity-based tracking and classification strategies as well as convolutional neuronal networks (CNN), we were unfortunately not yet able to automate the analysis to the level of accuracy presented by manual analysis. Fluorescence based imaging-techniques (with higher signal-to-noise ratios) might support the automation of classification but could on the other hand introduce bleaching artifacts and/or phototoxicity.

Establishing a causal link between precursor stability and branch stability could involve modifying precursor stability by pharmacological or genetical means. In this respect, the actin-stabilising functions of Gas2L1 and the pharmacological alteration of actin-stability in the same study could provide further insight (Willige et al., 2019). To stimulate such experiments, we have discussed the idea in lines 163-166.

3. The section in Statistical analysis states that Welch t-tests were used but does not address whether the data sets the parametric test was applied to were determined to be normal.

The assumptions of parametric statistical tests were tested using standard methods in statistical modelling: Normality of residuals was inspected by Q-Q plots, homoscedasticity by residuals-vs-fitted and scale-location plots, and susceptibility to outliers by Cook's distance plots (Line 291, previously line 223). We have additionally performed Shapiro-Wilk and Levene's test which confirm the results from previous assessments and added a corresponding statement to the methods section. Even when variances were not determined to differ between groups, we employed Welch's t-test (with Holm correction for multiple testing) to quantify statistical comparisons more robustly (Delacre et al., 2017).

4. As studies of branching usually do not clump immature dendrites and axons in the same data sets (if ever) but differentiate between the two types of processes, it is not clear to me why what is referred to as "location" (i.e., immature dendrites vs. axons) is considered a variable of value in the multifactorial analysis. A rationale for the inclusion of "location" in the multifactorial analysis, as it would apply other studies on branching, would benefit the reader.

Branching mechanisms of developing axons and dendrites are usually not studied in the same experiment based on the observed and described differences between the two mechanisms. We agree with this separation, in as far as treating branching data of axons and dendrites

© 2021. Published by The Company of Biologists under the terms of the Creative Commons Attribution License (https://creativecommons.org/licenses/by/4.0/).

may mislead conclusions about either process.

Studying both branching processes independently, however, precludes strong conclusions about <u>shared</u> processes of axonal and dendritic branching. Several studies describe dendritic branches to originate from the (morphologically) same collateral precursors that axon branches initiate from. While the molecular implementation differs, we noticed that precursor types have similar predictive capacity about branch stability on axons and dendrites. In other words, filopodia branches in our datasets are more long-lived than lamellipodia branches not only on axons, but also on immature dendrites.

Including the location (now termed 'neurite type') into a multifactorial analysis, allows a quantification of how different branches from the different neurites are and where they share similarities. Specific to our datasets, this allowed to distinguish two potential mechanisms in which developing axons influence their higher branch stability compared to dendrites: 1) axons predominantly initiate branches from the precursors that give rise to long-lived branches (filopodia and mixed); 2) axons stabilise branches even after normalising for the effect of these precursor types, indicating additional mechanisms for branch stabilisation independent from the precursor type system.

We have added a description and discussion of these reasons in lines 185-200.

5. As the importance of considering multifactorial causal analysis in cell biological studies is a major claim/conclusion of the work, I would suggest the presentation would greatly benefit for a much clearer exposition of the concept and method (as indeed most of us cell biologist are likely not knowledgeable about it, this was my first introduction to it). I would suggest two major revisions; (1) The introduction would greatly benefit from a clear paragraph presenting the analytical approach thereby priming the reader for what is to follow. (2) In the results, specifics about the analysis be presented to assist the reader in following how the information is extracted from the data sets using the analysis. (3) One aspect of the presentation that would benefit from revision is an early introduction to causal graphs. When Fig 1F is referenced, the reader is left to wonder what the significance of panel F may be. Indeed, it is only referenced as a cluster 1D-F and no specific attention is drawn to it in the text and the legend only provides a statement of fact about the panel; that it is a causal graph, but as a reader I had no idea what that meant or how it contributed. (4) a specific example: Line 105. Here again a clearer presentation of causal graph theory would greatly assist the reader. What is a "collider"? (4) Line 48 is the only mention of "hazard ratio" and line 236 the only mention of "Cox proportional hazards models", and Supp Fig 2 is referenced without any clear discussion/presentation. Here again, a naïve reader not familiar with the method is at a complete loss (I certainly was). In conclusion, the authors are urged to provide a primer for the readers regarding the analytic method being applied. It would really help a reader if they were "taken by the hand" and walked through the method step by step with rationales and justifications provided along the way.

We want to thank the reviewer for this excellent comment and, especially, for providing the specific examples. They have helped to pinpoint where the original description of the methodology was imprecise.

To (hopefully) resolve some of the questions, we want to start with one clarification: we employed three concepts that are uncommon in cell biology: 1) survival analysis to handle censored data; 2) multifactorial versions of a statistical model; 3) causal graph theory. These are all relevant for this specific study, but survival analysis is not essential for the methodological point of the discussion.

To clarify, we have outlined the rationale of the proposed and employed analysis strategy clearer in the results section and added an introductory Box 1 below (for potential use in the final article for minimal disruption of the presentation of biological results). We have tried our best to introduce the concepts as accurately and accessible as possible. However, we also have to acknowledge that the rationale behind these concepts accumulated over decades in related fields. It was, therefore, difficult to keep an introduction and justification of all mentioned topics explanatory <u>and</u> concise enough so the findings of the paper were not masked. For this reason, we focused on providing the basic facts summarising the topic and

© 2021. Published by The Company of Biologists under the terms of the Creative Commons Attribution License (https://creativecommons.org/licenses/by/4.0/).

refer to excellent resources that are both exhaustive and accessible to cell biologists.

Nevertheless, we want to address the specific comments and questions of the reviewer here in more detail:

(1) We have extended the description of the analytical approach and its rationale in the results section (lines 39-46, line 95-108) and in an additional Box 1 below this response where we introduce and connect concepts to the specific points in the manuscript and highlight relevant and accessible literature.

(2) We have added an explanation about the interpretation of the hazard ratio for this specific case of analysis and clearer connected the Cox-proportional hazard method with its umbrella survival analysis (lines 45-47).

(3) We included causal graphs in figures 1 & 2 in an attempt to make them more accessible; figures 1 and 2 show the smallest possible causal graphs linking only two factors. In this format readers could see them as building blocks of the causal graph presented in Fig. 3 when referring to the previous figures. We hope that Box 1, the referenced work by Judea Pearl and the fantastic course by Miguel Hernan (Hernan, 2017) can help to clarify any remaining questions.

(4) A collider is a specific configuration in a causal graph where two causal factors converge ('collide') on a third variable. Including such a variable into a statistical model can introduce so-called 'selection bias'. An illustrative example by Griffith et al. (2020) highlights this rather unintuitive feature of causal analyses: if universities select students either by academic or sporting ability, only top-performers in either category enrich in universities. As in the general population sporting and academic abilities are not correlated, athletic students have no specific predominance to be academically adept or vice versa. In the selected university sample, however, this selection procedure creates a spurious negative correlation of athletic and academic abilities. In the present study, the precursor type composition is both influenced by the PLPPR3 genotype as well as the neurite type - both factors 'collide' on precursor type. Therefore, to not introduce a spurious correlation on branch stability, also the neurite type has to be controlled for in an analysis. We believe that this specific explanation is, however, only peripheral to the main methodological argument of the paper, that multifactorial analyses can help to reduce the complexity in biological claims. We have, therefore, decided to exclude the corresponding phrase from the manuscript, as in this context it raises more questions than it answers.

(5) We have clarified the purpose of the Cox proportional hazard methodology and hazard ratios in the first paragraph of the results section. Figure S2 was included into Fig. 3 as requested by reviewer 1 and is a graphical representation of the hazard ratios reported in the results section to Fig. 3.

6. Lines 93-94. I do not follow what the authors mean when they write that " We showed that PLPPR3-loss decreases branch stability and reduces the numbers of the most efficient precursor (filopodia), preferentially on axons, which themselves stabilise branches in two distinct ways". In what two distinct ways do filopodia stabilize branches?

Thank you for detecting this ambiguity. The 'two distinct ways' were supposed to refer to axons (not filopodia), which appear to harbour more stable branches both by preferentially utilising the efficient precursors, as well as additional (direct) effects on stability.

We have now rephrased the respective sentence to 'We showed that PLPPR3-loss decreases branch stability and reduces the numbers of the most efficient precursor (filopodia). It does so preferentially on axons, which themselves stabilise branches and utilise more efficient precursors.'

© 2021. Published by The Company of Biologists under the terms of the Creative Commons Attribution License (https://creativecommons.org/licenses/by/4.0/).

In addition, we have included a clarification in the new discussion paragraph on commonalities and differences of axonal and dendritic branching (lines 185-200).

7. Line 115-116. "We identified that filopodia are not the most abundant precursor, but the most efficient". Lest I am misreading Fig 3A, filopodia account for approximately 50% of collateral branches, and the majority of the rest of the branching is through mixed filopodia-lamellipodia structures. The latter could also be argued to be argue to be mostly filopodial as the mixed are described as filopodia that then transition to having lamellipodial structures. Thus, it is not clear to me how the claim that filopodia are not the most abundant precursor.

Fig. 3A distinguishes axonal from dendritic branches as well as wild type from Plppr3$^{-/-}$. On axons (top row of Fig. 3A), filopodia are indeed the most abundant branch precursor type. On immature dendrites (bottom panel), however, they are hardly found, while collateral branches form mainly from lamellipodial structures. The statement refers to the comparison of overall branch initiations of a cell from all neurite types. This equality is visualised in the branch initiation quantification Fig. S1B (previously Fig. 3C).

8. The discussion of the possible cell biological underpinnings of the differences in branch stability between filopodia and lamellipodia formed branches may benefit from consideration of studies in embryonic sensory neurons, where it is my understanding the majority of branches arises from filopodia precursors. The base of filopodia along the axon contains septin-7 and drebrin, two proteins that assist in targeting microtubules into filopodia and early branches (Curr Biol. 2012 Jun 19;22(12):1109-15; Dev Neurobiol. 2016 Oct;76(10):1092-110) and these proteins are retained at the base of branches or are present shaft as they elongate. To my understanding neither septin-7 nor drebrin are known to mediate functions in lamellipodial actin networks. Thus, proteins such as septin-7 and drebrin may contribute to the greater stability of branches formed from filopodia. It would be interesting is these proteins were not found associated with the bases or shafts of branches that have arisen from lamellipodia, but at this point it's a discussion item.

We thank the reviewer for this excellent suggestion. These are indeed interesting candidate proteins that could mediate the observed effects and it would be very interesting to test their contributions in this analysis framework. We therefore have added a reference on septin-7 and drebrin with the corresponding papers to the discussion about potential mechanisms for branch stabilization of the precursor types.

9. Minor suggestion: the term "location" to differentiate whether a branch formed from an axon or an immature dendrite in the text and Fig 1F may not be the best choice of terminology as what is being differentiated are two different types of neuronal processes. Personally, upon reading about "location" I assumed the authors might refer to location along the axon shaft (e.g., closer or further from the cell body). Thus, I would suggest revising the terminology and switching from "location" to "type of neuronal process" or more directly "Axon vrs Immature Dendrite".

We absolutely agree that 'location' can be misleading, especially given the literature on distal versus proximal branching mechanisms. We have changed the term to 'neurite type' instead and now refer to developing axons as 'axons' and developing dendrites as 'immature dendrites' (instead of the equally ambiguous 'neurites').

Reviewer 3:

1. It will be interesting to better define the mixed precursor branches, and if after closer inspection and analysis of their behavior they can be assigned as finally originated from filopodia or lamellipodia. If not possible, then it will be important to discuss if the mechanisms associated to the formation of these mixed precursor branches is qualitatively different from the ones originating from filopodia or lamellipodia. Looking at survival in Fig.2C it seems that they behave more like a lamellipodia-associated branch.

The mixed branch precursors are indeed a very intriguing class in this dataset. Their lifetime ranges between that of filopodia and lamellipodia with a slight tendency towards the lamellipodia-associated branches in the datasets analysed. This suggests that lamellipodia

© 2021. Published by The Company of Biologists under the terms of the Creative Commons Attribution License (https://creativecommons.org/licenses/by/4.0/).

associated branch forming events might dominate the effects on branch stability and filopodia were just in the right place where the lamellipodium associated branch formed. Nevertheless, a large proportion of branches from 'mixed' precursors appear to persist longer than lamellipodial-associated branches (Fig. 2B), arguing that at least a subpopulation of these branches might be associated with filopodial effects.

All these interpretations, however, assume that 'mixed' branches are just an artificial class in our dataset that in fact is made up from filopodial and lamellipodial precursors - reflected also by our subjective naming scheme ('mixed' instead of an independent feature name). Indeed, one main rationale to include this category in the analysis was, to have a class for precursor types with ambiguous classification (similar to the 'unclear' class for neurite types), to not bias the estimation of the 'purer' other three precursor classes or the neurite-type and genotype comparisons. Our data therefore unfortunately provides little to no information on the question whether mixed branches are a mix of filopodia and lamellipodia events or whether they are an independent category.

We believe that the answer to this question lies in studies elucidating the molecular mechanistic differences in branch initiation and stabilization between filopodial and lamellipodial branches. Mixed precursors could as an example be scored for presence or absence of marker proteins for filopodia-branches. However, to our knowledge, the specific localization of most candidates on subsequent branches have not been specifically assessed yet.

2. It will be important to extend the discussion about multifactorial statistical analysis, in terms of the requirements for this type of analysis. As the author state, this type of analysis can really advance the definition of relationships in multifactorial experiments, and the readers will benefit form some details about their implementation.

We thank the reviewer for this key criticism. We have added an explanatory Box 1 below (for potential use in the final article) and added further explanations in the results section (lines 39-46, line 95-108), and highlighted literature accessible to biologists about the concept. We furthermore refer to our answer to comment 5 of Reviewer 2 on the same topic.

Box 1: Multifactorial analyses and causal graphs

A major purpose of statistical analyses is to estimate how strong and how reliable the levels of one variable associate with levels of another variable: how much is a knockout of *Plppr3* associated with short-lived branches, how likely are stable branches found on axons versus dendrites. Often, however, variables are associated with multiple other variables – e.g., the stability of a branch with the precursor-, neurite and genotype. Correcting for multiple sources of variance ("effects") in such data requires measuring (if possible even experimentally manipulating) the relevant sources of variance – in the same experiment. Quantifying the contributions of all variables requires analysing them in one model rather than with separate statistical tests, as illustrated in this study.

The associations of multiple variables with an outcome can be analysed (amongst other techniques) using multiple linear regression or ANOVA methodologies. Such multivariable or multifactorial models are more general cases of commonly used unifactorial statistical models: t-tests (one associated factor with two levels) are special cases of one-way ANOVA (one associated factor with more than two levels), which are special cases of two-way ANOVA (two associated factors). Linear regression (one associated variable) are special cases of multiple regressions (several associated variables). All of those techniques can be summarised under the umbrella term of 'general linear models'(Cohen, 1968). Also, survival analyses using the Cox proportional hazard methodology can be readily expanded to include multiple variables. For further introductions on the commonalities and the use of more general statistical models, we recommended Gelman et al., 2020, Harrell, 2015 and specifically for cell biology Lazic, 2016).

Statistical tools can, however, in principle only quantify associations – they are blind about the causal directions (Greenland et al., 1999; Pearl, 1995). As researchers, we use additional information (e.g., temporal information or interventions) to interpret a causal structure in these

© 2021. Published by The Company of Biologists under the terms of the Creative Commons Attribution License (https://creativecommons.org/licenses/by/4.0/).

associations. However, especially when several factors influence an outcome, causal interpretations are complicated by the interplay of all factors. Non-causal association can arise from common causes, which can easily be “controlled for” by estimating shared contributions of such confounders in a multifactorial analysis. However, it is not always advisable to include all potentially associated factors in a statistical model when studying causation: associations can be masked by controlling for mediators or be introduced by selecting on colliders (for a recent discussion see Griffith et al., 2020).

Causal diagrams (or causal graphs) can help to discern which variables remove or introduce bias in causal interpretations. They visualise research hypotheses and can be informed by the interpretation of data, as exemplified in Fig. 1C & F with the simplest possible causal graphs only establishing directionality of an association between two factors. The causal graph in Fig. 3C summarises the interpretation of data from figures 1-3. Such causal graphs can in turn help to de-confound specific statistical models. To estimate the strength of a specific connection in a causal graph without bias from other factors, the specific connection has to be “d-separated” from the other factors (Pearl et al., 2016). Applied to this study, to establish the strength of a direct effect of PLPPR3 on branch stability, both the effects of precursor types and neurite types have to be included in a multifactorial model. For detailed introductions into causal inference and its connection to statistics, we recommend: Hernan, 2017; Hernán and Robins, 2020; Pearl et al., 2016; Rohrer, 2018; Suttorp et al., 2015).

References

Brosig A, Fuchs J, Ipek F, Kroon C, Schrötter S, Vadhvani M, Polyzou A, Ledderose J, van Diepen M, Holzhütter HG, Trimbuch T, Gimber N, Schmoranzer J, Lieberam I, Rosenmund C, Spahn C, Scheerer P, Szczepek M, Leondaritis G, Eickholt BJ. 2019. The Axonal Membrane Protein PRG2 Inhibits PTEN and Directs Growth to Branches. *Cell Rep* **29**:2028-2040.e8. doi:10.1016/j.celrep.2019.10.039

Castro AF, Baltruschat L, Stürner T, Bahrami A, Jedlicka P, Tavosanis G, Cuntz H. 2020. Achieving functional neuronal dendrite structure through sequential stochastic growth and retraction. *Elife* **9**:1–38. doi:10.7554/eLife.60920

Cohen J. 1968. Multiple regression as a general data-analytic system. *Psychol Bull* **70**:426–443. doi:10.1037/h0026714

Delacre M, Lakens D, Leys C. 2017. Why psychologists should by default use welch’s t-Test instead of student’s t-Test. *Int Rev Soc Psychol* **30**:92–101. doi:10.5334/irsp.82

Dent EW, Kalil K. 2001. Axon branching requires interactions between dynamic microtubules and actin filaments. *J Neurosci* **21**:9757–69.

Flynn KC, Pak CW, Shaw AE, Bradke F, Bamburg JR. 2009. Growth cone-like waves transport actin and promote axonogenesis and neurite branching. *Dev Neurobiol* **69**:761–779. doi:10.1002/dneu.20734

Gallo G, Letourneau PC. 1999. Different Contributions of Microtubule Dynamics and Transport to the Growth of Axons and Collateral Sprouts. *J Neurosci* **19**:3860–3873. doi:10.1523/JNEUROSCI.19-10- 03860.1999

Gascon E, Dayer AG, Sauvain MO, Potter G, Jenny B, De Roo M, Zgraggen E, Demaurex N, Muller D, Kiss JZ. 2006. GABA regulates dendritic growth by stabilizing lamellipodia in newly generated interneurons of the olfactory bulb. *J Neurosci* **26**:12956–12966. doi:10.1523/JNEUROSCI.4508-06.2006

Gelman A, Hill J, Vehtari A. 2020. Regression and Other Stories. Cambridge University Press. doi:10.1017/9781139161879

Gibson DA, Ma L. 2011. Developmental regulation of axon branching in the vertebrate nervous

© 2021. Published by The Company of Biologists under the terms of the Creative Commons Attribution License (https://creativecommons.org/licenses/by/4.0/).

system. *Development* **138**:183–195. doi:10.1242/dev.046441

Greenland S, Pearl J, Robins JJM. 1999. Causal Diagrams for Epidemiologic Research. *Epidemiology* **10**:37–48. doi:10.1097/00001648-199901000-00008

Griffith GJ, Morris TT, Tudball MJ, Herbert A, Mancano G, Pike L, Sharp GC, Sterne J, Palmer TM, Davey Smith G, Tilling K, Zuccolo L, Davies NM, Hemani G. 2020. Collider bias undermines our understanding of COVID-19 disease risk and severity. *Nat Commun* **11**:1–12. doi:10.1038/s41467-020-19478-2

Harrell FE. 2015. Regression Modeling Strategies, Springer Series in Statistics. Cham: Springer International Publishing. doi:10.1007/978-3-319-19425-7

Hernan M. 2017. Causal Diagrams: Draw Your Assumptions Before Your Conclusions | edX. *edX*. https://www.edx.org/course/causal-diagrams-draw-your-assumptions-before-your

Hernán M, Robins J. 2020. Causal Inference: What If. Boca Raton: Chapman & Hall/CRC. Lazic SE. 2016. Experimental Design for Laboratory Biologists. Cambridge University Press. doi:10.1017/9781139696647

Leung KM, Elashoff RM, Afifi AA. 1997. Censoring issues in survival analysis. *Annu Rev Public Health* **18**:83–104. doi:10.1146/annurev.publhealth.18.1.83

Lewis TL, Courchet J, Polleux F. 2013. Cellular and molecular mechanisms underlying axon formation, growth, and branching. *J Cell Biol* **202**:837–848. doi:10.1083/jcb.201305098

Liu X, Huai J, Endle H, Schlüter L, Fan W, Li Y, Richers S, Yurugi H, Rajalingam K, Ji H, Cheng H, Rister B, Horta G, Baumgart J, Berger H, Laube G, Schmitt U, Schmeisser MJ, Boeckers TM, Tenzer S, Vlachos A, Deller T, Nitsch R, Vogt J. 2016. PRG-1 Regulates Synaptic Plasticity via Intracellular PP2A/β1-Integrin Signaling. *Dev Cell* 1–16. doi:10.1016/j.devcel.2016.06.019

Lowery LA, Van Vactor D. 2009. The trip of the tip: understanding the growth cone machinery. *Nat Rev Mol Cell Biol* **10**:332–343. doi:10.1038/nrm2679

Pearl J. 1995. Causal diagrams for empirical research. *Biometrika* **82**:669–688. doi:10.1093/biomet/82.4.669

Pearl J, Glymour M, Jewell NP. 2016. Causal Inference in Statistics: A Primer. Chichester, England: John Wiley & Sons, Inc.

Rohrer JM. 2018. Thinking Clearly About Correlations and Causation: Graphical Causal Models for Observational Data. *Adv Methods Pract Psychol Sci* **1**:27–42. doi:10.1177/2515245917745629

Santos TE, Schaffran B, Broguière N, Meyn L, Zenobi-Wong M, Bradke F. 2020. Axon Growth of CNS Neurons in Three Dimensions Is Amoeboid and Independent of Adhesions. *Cell Rep* **32**. doi:10.1016/j.celrep.2020.107907

Snider J, Pillai A, Stevens CF. 2010. A Universal Property of Axonal and Dendritic Arbors. *Neuron* **66**:45–56. doi:10.1016/j.neuron.2010.02.013

Suttorp MM, Siegerink B, Jager KJ, Zoccali C, Dekker FW. 2015. Graphical presentation of confounding in directed acyclic graphs. *Nephrol Dial Transplant* **30**:1418–1423. doi:10.1093/ndt/gfu325

Tilve S, Iweka CA, Bao J, Hawken N, Mencio CP, Geller HM. 2020. Phospholipid phosphatase related 1 (PLPPR1) increases cell adhesion through modulation of Rac1 activity, Experimental Cell Research. Elsevier Inc. doi:10.1016/j.yexcr.2020.111911

Tymanskyj SR, Ma L. 2019. MAP7 Prevents Axonal Branch Retraction by Creating a Stable

© 2021. Published by The Company of Biologists under the terms of the Creative Commons Attribution License (https://creativecommons.org/licenses/by/4.0/).

Microtubule Boundary to Rescue Polymerization. *J Neurosci* **39**:7118–7131. doi:10.1523/jneurosci.0775-19.2019

Van Beuningen SFB, Will L, Harterink M, Chazeau A, Van Battum EY, Frias CP, Franker MAM, Katrukha EA, Stucchi R, Vocking K, Antunes AT, Slenders L, Doulkeridou S, Sillevis Smitt P, Altelaar AFM, Post JA, Akhmanova A, Pasterkamp RJ, Kapitein LC, de Graaff E, Hoogenraad CC. 2015. TRIM46 Controls Neuronal Polarity and Axon Specification by Driving the Formation of Parallel Microtubule Arrays. *Neuron* **88**:1208–1226. doi:10.1016/j.neuron.2015.11.012

Westreich D, Greenland S. 2013. The table 2 fallacy: Presenting and interpreting confounder and modifier coefficients. *Am J Epidemiol* **177**:292–298. doi:10.1093/aje/kws412

Willige D, Hummel JJ, Alkemade C, Kahn OI, Au FK, Qi RZ, Dogterom M, Koenderink GH, Hoogenraad CC, Akhmanova A. 2019. Cytolinker Gas2L1 regulates axon morphology through microtubule-modulated actin stabilization. *EMBO Rep* **20**:1–20. doi:10.15252/embr.201947732

Witte H, Neukirchen D, Bradke F. 2008. Microtubule stabilization specifies initial neuronal polarization. *J Cell Biol* **180**:619–632. doi:10.1083/jcb.200707042

Yu W, Qiang L, Solowska JM, Karabay A, Korulu S, Baas PW. 2008. The Microtubule-severing Proteins Spastin and Katanin Participate Differently in the Formation of Axonal Branches. *Mol Biol Cell* **19**:1485–1498. doi:10.1091/mbc.e07-09-0878

Second decision letter

MS ID#: JOCES/2021/258983

MS TITLE: Precursor types predict the stability of neuronal branches

AUTHORS: Joachim Fuchs and Britta J Eickholt
ARTICLE TYPE: Research Article

I am happy to tell you that your manuscript has been accepted for publication in Journal of Cell Science, pending standard ethics checks.

Reviewer 2

Advance summary and potential significance to field

This paper uses statistical analytical methods to determine that the stability of axon branches is associated with the type of precursor structure from which the branch forms (i.e., filopodia vs lamellipodia). While not providing mechanistic insight into how this association arises, the basic determination is novel and of significance.

Comments for the author

The authors were responsive to reviews and have extensively modified the manuscript and generated new data sets, as suggested. This reviewer does not have any further concerns.

© 2021. Published by The Company of Biologists under the terms of the Creative Commons Attribution License (https://creativecommons.org/licenses/by/4.0/).

Reviewer 3

Advance summary and potential significance to field

The authors have answer the issues raised by this reviewer.

Comments for the author

No further revisions from this reviewer.

© 2021. Published by The Company of Biologists under the terms of the Creative Commons Attribution License (https://creativecommons.org/licenses/by/4.0/).
